# Artemisinin inhibits neutrophil and macrophage chemotaxis, cytokine production and NET release

**DOI:** 10.1038/s41598-022-15214-6

**Published:** 2022-06-30

**Authors:** Hassan O. J. Morad, Suaib Luqman, Larissa Garcia Pinto, Kevin P. Cunningham, Bruno Vilar, Georgia Clayton, Manu Shankar-Hari, Peter A. McNaughton

**Affiliations:** 1 Wolfson Centre for Age-Related Diseases, King’s College London, Guy’s Campus, London Bridge, London, SE1 1UL UK; 2 Bioprospection and Product Development Division, CSIR-Central Institute of Medicinal and Aromatic Plants, P.O. CIMAP, Lucknow, Uttar Pradesh 226015 India; 3 School of Immunology and Microbial Sciences, King’s College London, Guy’s Campus, London Bridge, London, SE1 1UL UK; 4 The Queen’s Medical Research Institute, Edinburgh BioQuarter, Centre for Inflammation Research, 47 Little France Crescent, Edinburgh, EH16 4TJ UK; 5 Department of Intensive Care Medicine, Royal Infirmary of Edinburgh, Edinburgh, UK

**Keywords:** Biophysics, Cell biology, Drug discovery, Immunology, Diseases, Medical research, Molecular medicine

## Abstract

Immune cell chemotaxis to the sites of pathogen invasion is critical for fighting infection, but in life-threatening conditions such as sepsis and Covid-19, excess activation of the innate immune system is thought to cause a damaging invasion of immune cells into tissues and a consequent excessive release of cytokines, chemokines and neutrophil extracellular traps (NETs). In these circumstances, tempering excessive activation of the innate immune system may, paradoxically, promote recovery. Here we identify the antimalarial compound artemisinin as a potent and selective inhibitor of neutrophil and macrophage chemotaxis induced by a range of chemotactic agents. Artemisinin released calcium from intracellular stores in a similar way to thapsigargin, a known inhibitor of the Sarco/Endoplasmic Reticulum Calcium ATPase pump (SERCA), but unlike thapsigargin, artemisinin blocks only the SERCA3 isoform. Inhibition of SERCA3 by artemisinin was irreversible and was inhibited by iron chelation, suggesting iron-catalysed alkylation of a specific cysteine residue in SERCA3 as the mechanism by which artemisinin inhibits neutrophil motility. In murine infection models, artemisinin potently suppressed neutrophil invasion into both peritoneum and lung in vivo and inhibited the release of cytokines/chemokines and NETs. This work suggests that artemisinin may have value as a therapy in conditions such as sepsis and Covid-19 in which over-activation of the innate immune system causes tissue injury that can lead to death.

## Introduction

An important cause of the deterioration leading to death in Covid-19 patients is thought to be an excessive release of pro-inflammatory cytokines from innate immune cells, which precipitates the severe lung condition named acute respiratory distress syndrome (ARDS)^1^. Other organs are also often affected in Covid-19, in a syndrome more akin to systemic sepsis^2^. ARDS in Covid-19 may therefore be attributable to overactivation of the innate immune system, leading to (i) neutrophil invasion of the lung^3^; (ii) excess release of pro-inflammatory cytokines^1,2^; and (iii) release of Neutrophil Extracellular Traps (NETs), sticky aggregations of DNA, histones and other proteins released from neutrophils^4^. In more localised infections, pro-inflammatory cytokines and chemokines perform a beneficial role, as chemoattractants that recruit immune cells to join the attack on invading pathogens, while NETs play a vital role in the physical snaring and immobilisation of pathogens. In system-wide sepsis, ARDS or Covid-19, however, excess cytokine, chemokine and NET release are thought to be critical in the events leading to clinical deterioration, organ dysfunction and death^1–3,5^.

In the present study we identified the natural compound artemisinin, which has achieved significant success as a front-line antimalarial, as a potent inhibitor of immune cell chemotaxis induced by a wide variety of chemoattractant molecules. We show that artemisinin suppresses neutrophil invasion in vivo and also successfully inhibits cytokine/chemokine secretion and NET release caused by a range of pro-inflammatory agents, including hydrogen peroxide (H_2_O_2_), bacterial lipopolysaccharide (LPS) and the SARS-CoV-2 spike protein. In view of the favourable clinical profile of artemisinin and its analogues, we suggest that these compounds may be useful as therapies in conditions such as systemic sepsis, ARDS and Covid-19.

## Results

### Artemisinin is a potent inhibitor of neutrophil and macrophage chemotaxis

**Figure 1:**
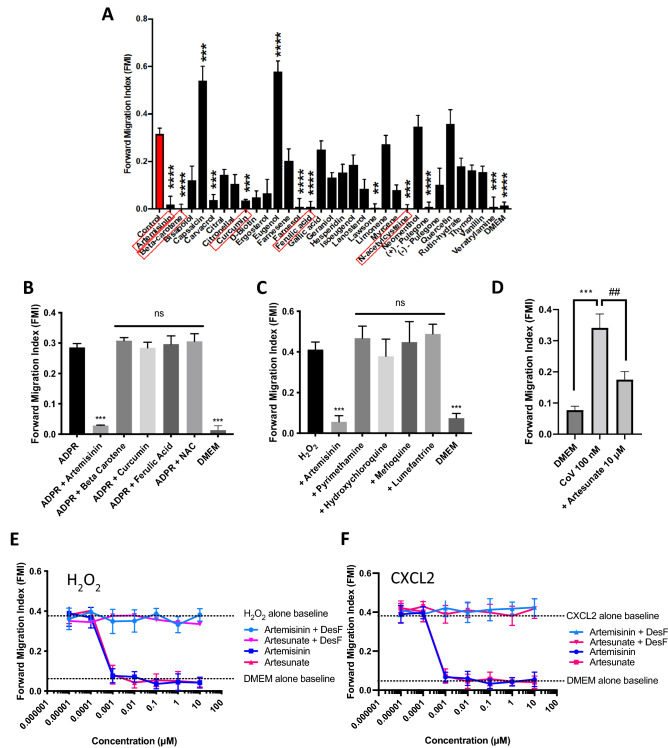
Artemisinin and its analogues are potent inhibitors of neutrophil chemotaxis. (**A**) Neutrophils migrated up a gradient of 10 nM H_2_O_2_ over 1 mm (see Methods). Forward migration index (FMI, vertical axis), the mean ratio of distance travelled in the direction of the chemoattractant gradient to total distance travelled, is a measure of chemoattraction (see details in ref^8^). Thirty-one compounds from a natural compound library were tested at 10 µM. Five compounds (red boxes) were selected on the basis that they caused the greatest inhibition of FMI, together with the greatest reduction in speed of movement (Supplementary Fig. 1A). Each bar shows mean ± SEM from n = 3 mice. Statistical analysis: For comparison with Control: **p < 0.01, ***p < 0.001, ****p < 0.0001 (One-way ANOVA and Tukey–Kramer post-hoc test). (**B**) Out of the five compounds inhibiting chemoattraction towards H_2_O_2_, only artemisinin inhibited migration up a gradient of ADPR, a direct activator of TRPM2. All neutrophils in each experiment from same batch; FMI in absence of inhibitor is consistent within batches but maximum value varies somewhat between batches. Each bar shows mean ± SEM from n = 3 mice. Statistical analysis: Comparison with ADPR alone: ***p < 0.001, ns = not significant. (One-way ANOVA and Tukey–Kramer post-hoc test). (**C**) Artemisinin (10 μM) completely inhibits neutrophil migration up a 10 nM gradient of H_2_O_2_ (FMI not significantly different from that in the absence of H_2_O_2_ gradient), while antimalarials pyrimethamine, hydroxychloroquine, mefluoquine and lumefantrine (all 10 μM) have no inhibitory effect. Each bar shows mean ± SEM from n = 3 mice. Comparison with H_2_O_2_: ***p < 0.001, ns = not significant. (One-way ANOVA and Tukey–Kramer post-hoc test). (**D**) Covid spike protein (SARS-CoV-2, 100 nM) is a potent neutrophil chemoattractant and chemoattraction is inhibited by artesunate (10 μM). Comparison with DMEM: ***p < 0.001, comparison with CoV + artesunate: ##p < 0.01 (One-way ANOVA and Tukey–Kramer post-hoc test). (**E**) Dose–response relation for inhibition of neutrophil FMI up a gradient of 10 nM H_2_O_2_ by artemisinin and its analogue artesunate. Dotted lines show mean value of FMI in gradient of H_2_O_2_ (upper) or in no chemoattractant (DMEM, lower). IC_50_ values: artemisinin, 0.36 nM; artesunate, 0.37 nM**.** No inhibition observed in presence of Fe^2+^ chelator desferrioxamine (DesF, 50 μM). Each point shows mean ± SEM from n = 3 mice. FMI in artemisinin /artesunate + DesF is significantly lower than artemisinin/artesunate values at all concentrations > 0.1 nM (p < 0.001). (**F**) Similar experiment to A, carried out with neutrophils in gradient of chemokine CXCL2 (10 nM). IC_50_ values: artesunate, 0.34 nM; artemisinin, 0.31 nM. Effect of artemisinin and artesunate completely abolished by desferrioxamine (50 μM). Each point shows mean ± SEM from n = 3 mice. FMI in artemisinin + DesF/artesunate + DesF is significantly lower than artemisinin/artesunate values at all concentrations > 0.1 nM (p < 0.001).

Hydrogen peroxide (H_2_O_2_) is known to act as a potent immune cell chemoattractant^6,7^. In previous work we have shown that the TRPM2 ion channel, which is activated by H_2_O_2_, mediates the chemotactic action of H_2_O_2_ by preferentially inducing a calcium influx at the neutrophil leading edge^8^. We initially searched for inhibitors of neutrophil chemotaxis by screening a natural compound library, using neutrophil chemotaxis towards H_2_O_2_ as the assay. Figure 1A shows the forward migration index (FMI), the ratio of linear distance travelled in the direction of the H_2_O_2_ gradient to the total distance travelled, which gives an index of the directionality of cell movement. Interestingly, capsaicin, a TRPV1 agonist^9^ and eugenol, a TRPV3 agonist^10^ both significantly *potentiated* directional chemotaxis, perhaps because they have a weak agonist action at TRPM2. Of the compounds that caused an inhibition of chemotaxis, five were identified as interesting for further investigation (red boxes in Fig. 1A), based on a significant reduction in FMI together with a significant reduction in average speed of migration (Supplementary Fig. 1A). The dose–response relations of four of these compounds, beta-carotene, curcumin, ferulic acid and N-acetylcysteine, were similar, suggesting a common action, while artemisinin was more potent (Supplementary Fig. 1B). The four compounds showing a similar potency are antioxidants, so we investigated the possibility that they may act indirectly by dissipating the gradient of H_2_O_2_. To test this idea, we used a gradient of adenosine diphosphate ribose (ADPR), which like H_2_O_2_ is a potent neutrophil chemoattractant^8,11^. ADPR directly activates TRPM2 at an intracellular location^12–14^, while H_2_O_2_ does not directly activate TRPM2 but acts by increasing intracellular levels of ADPR^13,14^. When a gradient of ADPR was used to activate neutrophil chemotaxis none of the four antioxidant compounds was able to inhibit chemotaxis (Fig. 1B), showing that their action was indeed to dissipate the H_2_O_2_ gradient rather than to directly inhibit chemotaxis. Artemisinin, on the other hand, inhibited neutrophil chemotaxis towards both ADPR and H_2_O_2_ (Fig. 1B,C, respectively), demonstrating that its action in abolishing chemotaxis is independent of any effect on the gradient of H_2_O_2_. We next compared the ability of artemisinin to inhibit neutrophil chemotaxis with that of other well-established antimalarial compounds. Artemisinin was the only antimalarial that inhibited neutrophil migration (Fig. 1C) and therefore has a unique mechanism of action. We found that artemisinin has no effect on neutrophil viability, ruling out the possibility of a toxic action of artemisinin as a basis for its inhibition of neutrophil chemotaxis (Supplementary Fig. 2). The SARS-CoV-2 spike protein was also a potent chemoattractant for neutrophils in our in vitro assay, and artemisinin also strongly inhibited chemotaxis up a gradient of SARS-CoV-2 (Fig. 1D).

Figure 1E shows that artemisinin and artesunate (an artemisinin analogue) are both highly potent inhibitors of neutrophil chemotaxis driven by H_2_O_2_, with IC_50_ ≈ 0.3 nM. Artemisinin and artesunate also strongly inhibit chemotaxis towards a diverse range of other chemotactic signals, including the chemokine CXCL2 (Fig. 1E), the complement factor C5a and the bacterial cell wall component lipopolysaccharide (LPS) (Supplementary Fig. 3). In each case, the values of IC_50_ for artemisinin and artesunate were close to 0.3 nM, with none being significantly different from the value obtained for chemotaxis towards H_2_O_2_. Supplementary Fig. 4 shows that macrophage chemotaxis was also potently inhibited by artemisinin and artesunate, in a similar way to the effects of these compounds on neutrophils. This work identifies artemisinin as a potent inhibitor of neutrophil and macrophage chemotaxis driven by a wide variety of chemoattractant agents.

### Mechanism of inhibition of chemotaxis by artemisinin

A number of active analogues of artemisinin have been developed for use as antimalarials, including arteether, artemether and artesunate (structures shown in Supplementary Fig. 5). All are rapidly metabolised in vivo to dihydroartemisinin (DHA), a more metabolically stable analogue with a longer in vivo half-life (~ 1.3 h) than any of its precursors^15,16^. All of these analogues, including the stable metabolite DHA, showed an equally high potency in inhibiting neutrophil chemotaxis towards a range of chemotactic signals (Fig. 1E,F and Supplementary Fig. 3; IC_50_ ≈ 0.3 nM for all analogues). These experiments show that none of the chemical modifications in these artemisinin analogues impacts on a site critical for the inhibitory action of artemisinin on chemotaxis.

**Figure 2:**
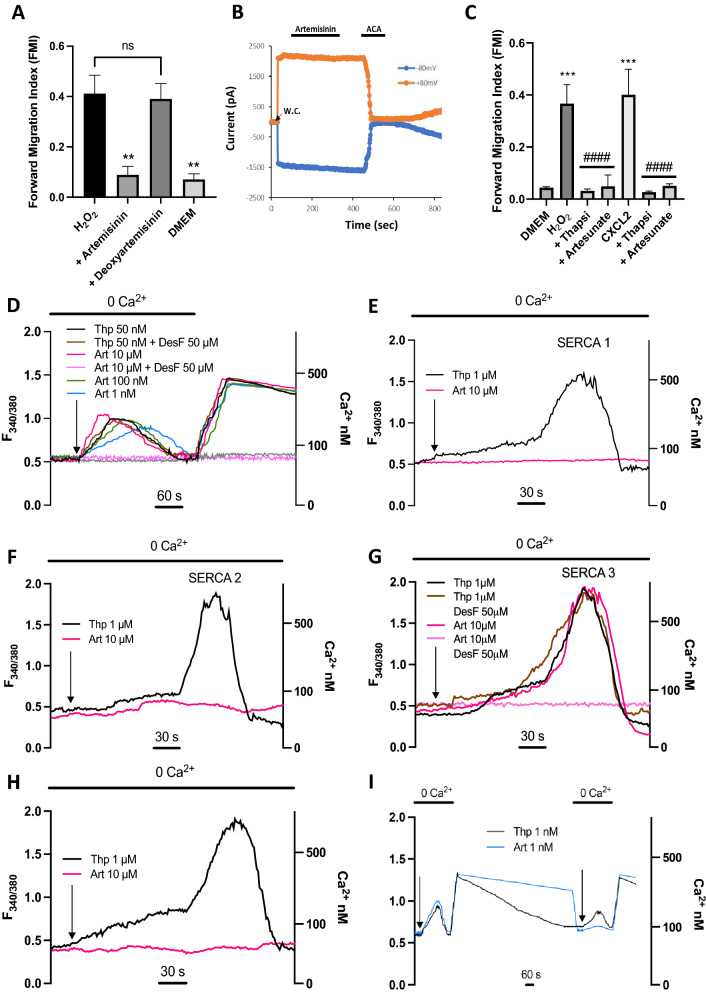
Mechanism of inhibition of neutrophil chemotaxis by artemisinin and analogues. (**A**) Inhibition of neutrophil chemotaxis by artemisinin (10 μM, second column) is completely abolished by removal of the peroxide bond in artemisinin (deoxyartemisinin, 10 μM, third column). Final column shows FMI in absence of gradient of H_2_O_2_. Each bar shows mean ± SEM from n = 3 mice. Statistical analysis: For comparison with H_2_O_2_: **, *p* < 0.01; ns = not significant. (One-way ANOVA and Tukey–Kramer post-hoc test). (**B**) Artemisinin does not block TRPM2 ion channels. Patch clamp recording of membrane current from TRPM2-transfected HEK293 cell at + 80 mV (orange) and − 80 mV (blue); TRPM2 ion channels activated by the inclusion of 1 mM ADPR in the intracellular patch clamp solution. Moment of breaking through to whole-cell mode shown by arrow. Artemisinin (10 μM) has no effect on membrane current (fractional current change 0.99 ± 0.04 at − 80 mV, 0.97 ± 0.03 at + 80 mV, neither significantly different from 1.0, n = 6), while the known TRPM2 inhibitor N-(p-amylcinnamoyl) anthranilic acid (ACA, 20 μM) suppresses membrane current at both membrane voltages (fractional current change 0.02 ± 0.01 at − 80 mV, 0.06 ± 0.01 at + 80 mV, both significantly different from 1.0, p < 0.0001, n = 6). (**C**) Neutrophil forward migration index (FMI) in a gradient of H_2_O_2_ (10 nM, bar 2) and CXCL2 (10 nM, bar 5) is abolished by artesunate (10 μM) and by the selective SERCA inhibitor thapsigargin (50 nM). Each bar shows mean ± SEM from n = 4 experiments with neutrophils from 4 mice. Statistics: ***, p < 0.001 compared to DMEM control; ####, p < 0.0001 compared to H_2_O_2_ or CXCL2. 0.001, one-way ANOVA and Tukey–Kramer post-hoc test. (**D**) Application of SERCA inhibitor thapsigargin (black trace, 50 nM) to a neutrophil releases calcium from intracellular stores (ratio measurement with fura-2, see Methods). Calcium influx from external medium prevented with 0Ca^2+^/2 mM EGTA (application time shown by bar at top). Similar dose-dependent release of intracellular store calcium seen with artemisinin (pink, 10 μM; green, 100 nM; blue, 1 nM) showing that artemisinin is a SERCA inhibitor. Increase of calcium on readmission of external Ca^2+^ is due to activation of store-operated calcium entry (SOCE) following store discharge and is similar in all cases, showing that artemisinin does not affect SOCE. Calcium release by artemisinin (10 μM) is inhibited by Fe^2+^ chelator desferrioxamine (DesF, light pink, 50 μM), but DesF has no effect on calcium release by thapsigargin (brown, 50 nM). Grey trace: no application of thapsigargin or artemisinin. Left axis: fura2 fluorescence ratio; right axis: [Ca^2+^]_i_ determined as in Methods. Example traces shown in **D**–**I** are typical of n = 7–12 cells imaged per cover slip, each condition repeated on 3 cover slips. (**E**) Similar experiment performed on HEK293 cell transfected with rSERCA1a. Calcium is released by thapsigargin (black, 1 $\mu$M) but not by artemisinin (pink, 10 μM). Other conditions as in D. (**G**) Similar experiment with overexpressed hSERCA2a. (**G**) Similar experiment with overexpressed hSERCA3. In contrast to SERCA1 and 2, artemisinin (pink, 10 μM) gives a similar calcium release to thapsigargin (black, 1 M$\mu$). Effect of artemisinin (10 μM) is completely inhibited by chelation of Fe^2+^ with desferrioxamine (light pink, 50 μM), while calcium release by thapsigargin is unaffected by desferrioxamine (brown, 50 μM). (**H**) Similar experiment on an untransfected HEK293 cell. Calcium stores are released by thapsigargin (black, 50 nM) but not by artemisinin (pink, 10 μM), showing that HEK293 cells, unlike neutrophils, do not express SERCA3. (**I**) Reversibility of inhibition of SERCA3 in neutrophils by thapsigargin but not artemisinin. Experimental protocol shown in D repeated twice with 21 min gap between applications. SOCE following 1 nM thapsigargin recovered and on readmission of 1 nM thapsigargin a second release of calcium from subcellular stores was observed. By contrast, SOCE following application of artemisinin (1 nM) diminished little and only a very small increase of calcium was observed on reapplication, showing that artemisinin is largely irreversible on the time scale tested here.

An unusual feature of artemisinin is the endoperoxide 1,2,4-trioxane ring (top left in Supplementary Fig. 5). We found that deoxyartemisinin, which lacks the peroxide bridge but is otherwise identical to artemisinin, is completely inactive in inhibiting neutrophil chemotaxis (Fig. 2A), showing that the presence of the peroxide bridge is essential for the action of artemisinin on chemotaxis. The critical role of the peroxide suggests that artemisinin may inhibit its protein target by oxidation. It has been known for many years that hydrogen peroxide can oxidise the sulfhydryl group in cysteine, and that this reaction depends on free ferrous iron^17^. We therefore investigated whether the action of artemisinin on neutrophil chemotaxis also depends on iron. Removing ferrous iron with the specific chelator desferrioxamine completely abrogated the ability of both artemisinin and artesunate to inhibit neutrophil chemotaxis at all concentrations (Fig. 1E,F). Antagonism by desferrioxamine of the inhibition of chemotaxis by artemisinin was independent of whether H_2_O_2_, a chemokine, C5a or lipopolysaccharide (LPS) were used as the chemoattractant (Supplementary Fig. 6). These observations suggest that artemisinin and its active derivatives may inhibit their protein target not by reversible antagonist binding, as has previously been supposed^18,19^, but instead by covalent modification of a cysteine residue, catalysed by Fe^2+^. Artemisinin and its analogues have been shown to be capable of alkylating both cysteine itself^20^ and the central cysteine residue in a cysteine-containing tripeptide, glutathione^21^, by oxidising and combining with the cysteine sulfhydryl (Supplementary Fig. 5B, C).

### SERCA is the cellular target of artemisinin

Neutrophil chemotaxis depends on the ability of chemoattractants to generate leading-edge calcium “pulses” that determine the direction of cell migration^8^. Supplementary Video 1 shows the generation of calcium pulses in a neutrophil migrating up a gradient of H_2_O_2_ (left-hand video), and the complete suppression of calcium pulses, together with chemotaxis, in the presence of artemisinin (second-left video). In the presence of a gradient of ADPR, calcium pulses drive chemotaxis in a similar way to H_2_O_2_, and artemisinin also inhibits both calcium pulses and chemotaxis (pair of videos on right). These experiments suggest that artemisinin prevents chemotaxis by inhibiting the generation of leading-edge calcium pulses.

In previous work we showed that chemotaxis driven by H_2_O_2_ depends on activation of the TRPM2 ion channel^8^. The importance of a calcium influx via TRPM2 for chemotaxis driven by H_2_O_2_ is shown in Supplementary Fig. 7. In this experiment, neutrophils were loaded with the calcium chelator BAPTA, which completely suppressed the intracellular calcium increase caused by activation of TRPM2 by H_2_O_2_ (Supplementary Fig. 7A). In the absence of this TRPM2-mediated calcium increase, neutrophil chemotaxis towards H_2_O_2_ was abolished (Supplementary Fig. 7B).

We next carried out patch-clamp experiments on TRPM2 heterologously expressed in HEK293 cells in order to test whether artemisinin might inhibit chemotaxis by blocking TRPM2. TRPM2 was activated by alternate positive and negative voltage pulses (Fig. 2B). Artemisinin had no significant effect on the current carried by TRPM2, in contrast to the known TRPM2 blocker ACA, which caused prompt and near-complete current inhibition.

A second reason for discarding TRPM2 as a target is that artemisinin inhibits, with equal potency, chemotaxis towards H_2_O_2_, ADPR (Fig. 1B,C) and a range of other chemoattractants (Fig. 1E,F and Supplementary Fig. 3). Chemotaxis activated by H_2_O_2_ and ADPR depends on activation of TRPM2^8^, but chemotaxis activated by cyto/chemokines depends on a separate pathway not involving TRPM2^8^. The schematic diagram in Supplementary Fig. 12 (steps 1–4) shows how H_2_O_2_ activates calcium influx through TRPM2, which in turn generates leading-edge calcium “pulses” that steer chemotaxis^8^. Leading-edge calcium pulses generated by cyto/chemokines and chemoattractants such as LPS^8^, on the other hand, depend on a separate pathway independent of TRPM2 (Supplementary Fig. 12, steps 6, 7). The ability of artemisinin to inhibit chemotaxis activated by each of these two distinct pathways implies that the action of artemisinin must be at a point common to both pathways, such as the sarcoplasmic and endoplasmic reticulum calcium ATPase (SERCA) that is responsible for refilling subcellular calcium stores, or the store-operated calcium entry mechanism (SOCE), that mediates calcium entry and store refilling following discharge of subcellular stores^22^. Both SERCA and SOCE have been shown to be functional in neutrophils^23^.

Thapsigargin, a potent and selective SERCA blocker^24^, completely inhibited neutrophil chemotaxis towards both H_2_O_2_ and the chemokine CXCL2, in a similar way to the inhibition caused by artemisinin (Fig. 2C), consistent with the idea that both thapsigargin and artemisinin exhaust the internal calcium stores that are necessary to drive chemotaxis^8^. Thapsigargin evoked an increase in internal calcium concentration in neutrophils in the complete absence of external calcium (Fig. 2D, black trace), that must be due to release from internal stores because no calcium influx across the surface membrane is possible. The calcium release was followed by a return to baseline levels as cytoplasmic calcium was extruded by surface membrane calcium pumps. When intracellular calcium stores had been exhausted, readmission of external calcium caused a sustained calcium increase, attributable to store-operated calcium entry (SOCE) carried via activation of calcium-selective Orai channels in the surface membrane^25^. The protocol shown in Fig. 2D therefore shows a way of separating a potential inhibitory action of artemisinin on SERCA and on SOCE.

Artemisinin evoked a dose-dependent increase in neutrophil intracellular calcium similar to that seen with thapsigargin (Fig. 2D), showing that artemisinin, like thapsigargin, acts to release calcium from intracellular stores of neutrophils and therefore may be a SERCA inhibitor. Artemisinin was effective down to a concentration of 1 nM in releasing calcium from intracellular stores, consistent with the high potency of artemisinin in inhibiting neutrophil chemotaxis (IC_50_ ≈ 0.3 nM, Fig. 1E,F). The intracellular calcium release evoked by thapsigargin was unaffected by the Fe^2+^ chelator desferrioxamine, but calcium release by artemisinin was completely suppressed (Fig. 2D), results that echo the effect of Fe^2+^ chelation on chemotaxis (Fig. 1E,F). At all concentrations of artemisinin, the profile of SOCE following readmission of calcium was similar to that caused by thapsigargin, showing that artemisinin does not interact with SOCE. These experiments are consistent with SERCA being the downstream target of artemisinin in neutrophils.

### Artemisinin irreversibly inhibits SERCA3

Thapsigargin is toxic to mammals^26^, while artemisinin has an excellent clinical safety record as an antimalarial, a difference that could arise from selective inhibition by artemisinin of a non-critical mammalian SERCA isoform, in contrast to the known ability of thapsigargin to inhibit all three SERCA isoforms equally^24^. SERCA1 is critical for muscle contraction, while SERCA2 is widely expressed in many essential organs^27^. Inhibition of either isoform would therefore be likely to cause significant toxicity. SERCA3, on the other hand, has a more limited expression pattern, which includes expression in immune cells^27^. These considerations suggest that SERCA3 may be the target of artemisinin in neutrophils.

In Fig. 2E–G we overexpressed mammalian SERCA1, 2 or 3 in HEK293 cells and then used the protocol shown in Fig. 2D to test for SERCA inhibition by thapsigargin or artemisinin. Thapsigargin released calcium from intracellular stores with a similar time course when applied to all SERCA isoforms, consistent with its ability to inhibit all isoforms equally^24^. Artemisinin, on the other hand, was inactive on cells transfected with SERCA1 and 2 (Fig. 2E,F) but released calcium with a similar time course to thapsigargin in cells transfected with SERCA3 (Fig. 2G). Removal of Fe^2+^ with desferrioxamine did not affect the ability of thapsigargin to inhibit SERCA3, but completely prevented inhibition of SERCA3 by artemisinin (Fig. 2G). Thapsigargin released calcium from intracellular stores of naïve HEK293 cells but artemisinin did not (Fig. 2H), consistent with expression of SERCA2 in HEK293 cells, that are derived from the kidney, where SERCA2 is the principal isoform^27^. As shown in Fig. 2D, artemisinin releases calcium from intracellular stores of neutrophils, consistent with the known expression of SERCA3 in cells of the immune system^27^. These experiments show that SERCA3 is the mammalian target of artemisinin.

Thapsigargin inhibits SERCA isoforms by binding reversibly to a location between membrane-spanning helices 3 and 7, deep within the motile machinery of the calcium pump^28^. The experiments above show that inhibition of SERCA3 by artemisinin depends, on the other hand, on its unusual peroxide bond, not present in thapsigargin, together with the presence of Fe^2+^ as a probable catalyst, suggesting a different mechanism involving irreversible covalent binding, likely to a cysteine residue. In Fig. 2I we used the protocol shown in Fig. 2D to compare the reversibility of SERCA inhibition by thapsigargin and artemisinin. Following exhaustion of calcium stores by thapsigargin, and consequent calcium influx via SOCE on readmission of external calcium, the intracellular calcium level returned slowly to its normal level over the 20 min following removal of thapsigargin, showing that SERCA had reactivated and intracellular stores had refilled, thus switching off SOCE. Readmission of thapsigargin again released calcium from intracellular stores, followed by reactivation of SOCE when extracellular calcium was readmitted, confirming the reversibility of thapsigargin binding to SERCA. However, when the same experiment was repeated using artemisinin, elevated calcium levels due to activation of SOCE persisted after store discharge, showing that stores had not refilled and that SERCA3 inhibition had therefore been maintained. On reapplying artemisinin in zero calcium, very little calcium release was observed, consistent with the lack of store refilling (Fig. 2I). This experiment confirms that inhibition of SERCA3 by artemisinin is essentially irreversible on the time scale used, in contrast to the reversible inhibition by thapsigargin.

### Artemisinin and analogues suppress in vivo neutrophil invasion in response to H_2_O_2_

**Figure 3:**
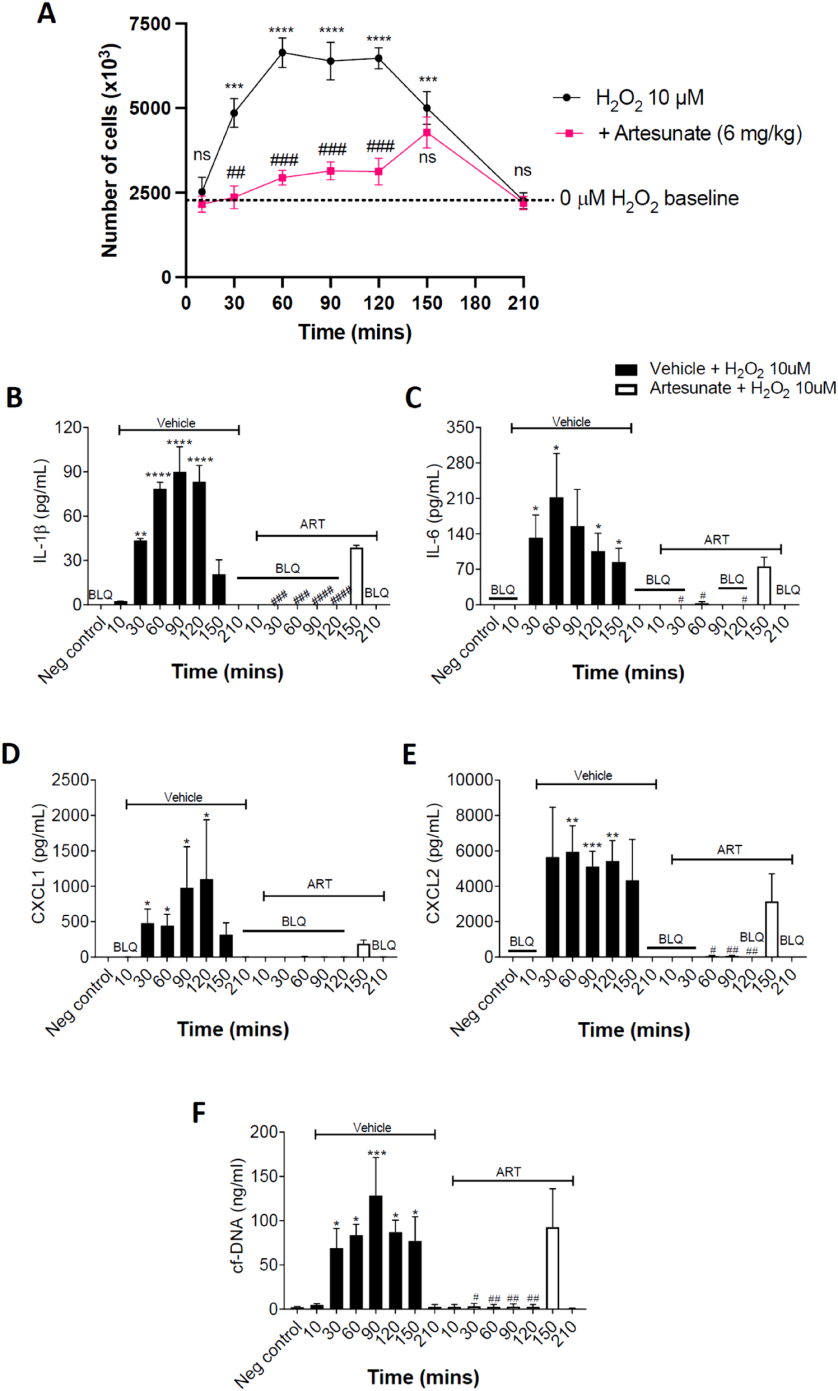
Artesunate suppresses neutrophil invasion and release of cytokines and NETs in response to intraperitoneal infusion of H_2_O_2_. (**A**) Number of cells recovered from intraperitoneal lavage following i.p. injection of H_2_O_2_ (10 µM in PBS, 10 μl/gm body weight). H_2_O_2_ causes a large influx of neutrophils that peaks at 60 min and reverses by 210 min (black points). Cells present in peritoneum before injection of H_2_O_2_ are tissue-resident macrophages, while cells entering the peritoneum following injection of H_2_O_2_ are neutrophils (see Methods for cell identification). Artesunate (6 mg/kg s.c., delivered 30 min before injection of H_2_O_2_) suppresses neutrophil influx for > 120 min (red points). Each point shows mean ± SEM from n = 4 mice. (**B**) Concentration of IL-1β in peritoneal lavage measured by ELISA, using i.p. lavage samples obtained as in A. Black bars show increase as function of time (mins) in vehicle-injected mice; open bars are corresponding data for mice injected with artesunate 6 mg/kg s.c., 30 min before injection of H_2_O_2_ as in A. Each bar shows mean ± SEM from n = 3 mice. (**C**,**D**,**E**) Similar data for IL-6, CXCL1 and CXCL2, obtained from same samples. (**F**) Similar data for release of NETs, quantified using Pico-Green kit. Statistical analysis: BLQ, below limit of quantitation; *, p < 0.05; **, P < 0.01; ***, p < 0.001, ****, p < 0.0001 compared with negative control (no H_2_O_2_); #, p < 0.05; ##, p < 0.01; ###, p < 0.001; ####p < 0.0001 artesunate group compared with no-artesunate group at same time point. ANOVA with Bonferroni post-hoc correction.

The potent action of artemisinin and its analogues in suppressing neutrophil chemotaxis in vitro suggests that these compounds may have a similar action in vivo, and therefore may potentially be useful as therapeutics in conditions such as ARDS and Covid-19 where excess immune cell invasion is an important driver of the pathology. We measured neutrophil invasion into mouse peritoneum following intraperitoneal injection of 10 μM H_2_O_2_, a concentration that we have found in previous work to have a maximal effect in activating neutrophil chemotaxis *in vitro*^8^. The time course of neutrophil invasion in response to i.p. H_2_O_2_ is shown in Fig. 3A. In this experiment total cell counts are shown; the background level of c. 2 × 10^6^ cells (lower dotted line) is attributable to the presence of tissue-resident macrophages^8^. Following injection of H_2_O_2_, neutrophil invasion causes the cell count to rise rapidly, reaching a peak of 6.5 × 10^6^ cells at 60 min, a level that is maintained until 120 min, followed by a return to baseline over the a further 90 min. Injection of artesunate s.c. 30 min prior to injection of H_2_O_2_ largely suppressed the neutrophil invasion up to 120 min, at which time the effect diminishes owing to the short in vivo lifetime of artesunate and its active metabolite dihydroartemisinin^15,16^. In agreement, Supplementary Fig. 8 shows that 10 μM H_2_O_2_ i.p. strongly activated an influx of neutrophils, and that neutrophil invasion was largely suppressed by injections of either artemisinin or artesunate at 28 mg/kg s.c., 30 min prior to injection of H_2_O_2_, with a slightly lesser effect at 6 mg/kg, a dose close to a typical clinically-used dose for artesunate of 2.4 mg/kg i.v. The similar in vivo inhibition by artemisinin and artesunate mirrors the similar actions of these two analogues in inhibiting neutrophil chemotaxis in vitro (Fig. 1E,F).

Excess release of cytokines/chemokines is thought to be critical in the pathology of conditions such as Covid-19 in which immune cell invasion plays an important role^1,2^. In Fig. 3B–E we used ELISA to measure the concentration of two pro-inflammatory cytokines, IL-1β and IL-6, and two chemokines, CXCL1 and CXCL2. In each case, the profile of increase following injection of H_2_O_2_ is similar to the profile of neutrophil invasion, rising from a low level to a broad peak at 60–120 min, followed by a return to undetectable levels by 210 min, a time at which the level of invading neutrophils had declined back to baseline. The suppression caused by prior injection of artesunate is striking, with the cytokine/chemokine increase near-completely abolished in all cases up to 120 min. An increase is seen at 150 min, in line with the recovery of neutrophil chemotaxis as the effect of artesunate wears off (Fig. 3A).

The release of neutrophil extracellular traps (NETs) from neutrophils may also augment the damaging effect of cytokines^3,5,29^. In Fig. 3F we examined the release of NETs by assaying cell-free DNA release. The profile is broadly similar to the release of cytokines; NET release shows a broad peak at 30–150 min, followed by a decline to low levels by 210 min as neutrophil invasion reverses. Artesunate completely inhibits NET release at times earlier than 150 min. An alternative assay of NET release using fluorescence microscopy showed a similar increase in NETs in response to in vitro application of LPS, also abolished by artemisinin (Supplementary Fig. 9).

A similar experiment carried out with infusion of H_2_O_2_ into the lung shows that neutrophil invasion, cytokine and chemokine release and NET release are all strongly suppressed by artesunate, as was found in the peritoneum (Supplementary Fig. 10). In summary, the release of cyto/chemokines and NETs in response to H_2_O_2_ parallels neutrophil invasion in both peritoneum and lung, and the ability of artesunate to inhibit neutrophil invasion has a striking effect in preventing the release of proinflammatory cytokines/chemokines and NETs.

### Artemisinin and analogues suppress in vivo neutrophil invasion in response to LPS and SARS-CoV-2 spike protein

Lipopolysaccharide (LPS), a constituent of the cell wall of gram-negative bacteria, plays a critical role in the interactions of many bacterial pathogens with the innate immune system^30^. LPS is a potent neutrophil chemoattractant in vitro*,* inducing chemotaxis that is steered by leading-edge calcium pulses^8^. The pathway by which LPS induces neutrophil chemotaxis is different from that activated by H_2_O_2_, however, because genetic deletion or pharmacological block of TRPM2 does not suppress chemotaxis towards LPS^8^ (see pathway diagram in Supplementary Fig. 12). In the experiment shown in Supplementary Fig. 11, we tested the ability of LPS to induce invasion of neutrophils into the peritoneum and the effect of artemisinin on this invasion. Neutrophil invasion into the peritoneum in response to LPS was activated more slowly than that induced by H_2_O_2_, so we sampled invasion at 5 h, and gave three doses of artemisinin s.c. at intervals of 2 h to maintain systemic levels of artemisinin throughout this time. LPS activated a neutrophil invasion that was similar in magnitude to that induced by H_2_O_2_, and the invasion was also largely suppressed by artemisinin (Supplementary Fig. 11A). The production of cytokines IL1-β and IL-6 and chemokines CXCL1 and CXCL2 was also strongly suppressed by artemisinin (Supplementary Fig. 11B-E), as was NET release (Supplementary Fig. 11F).

**Figure 4:**
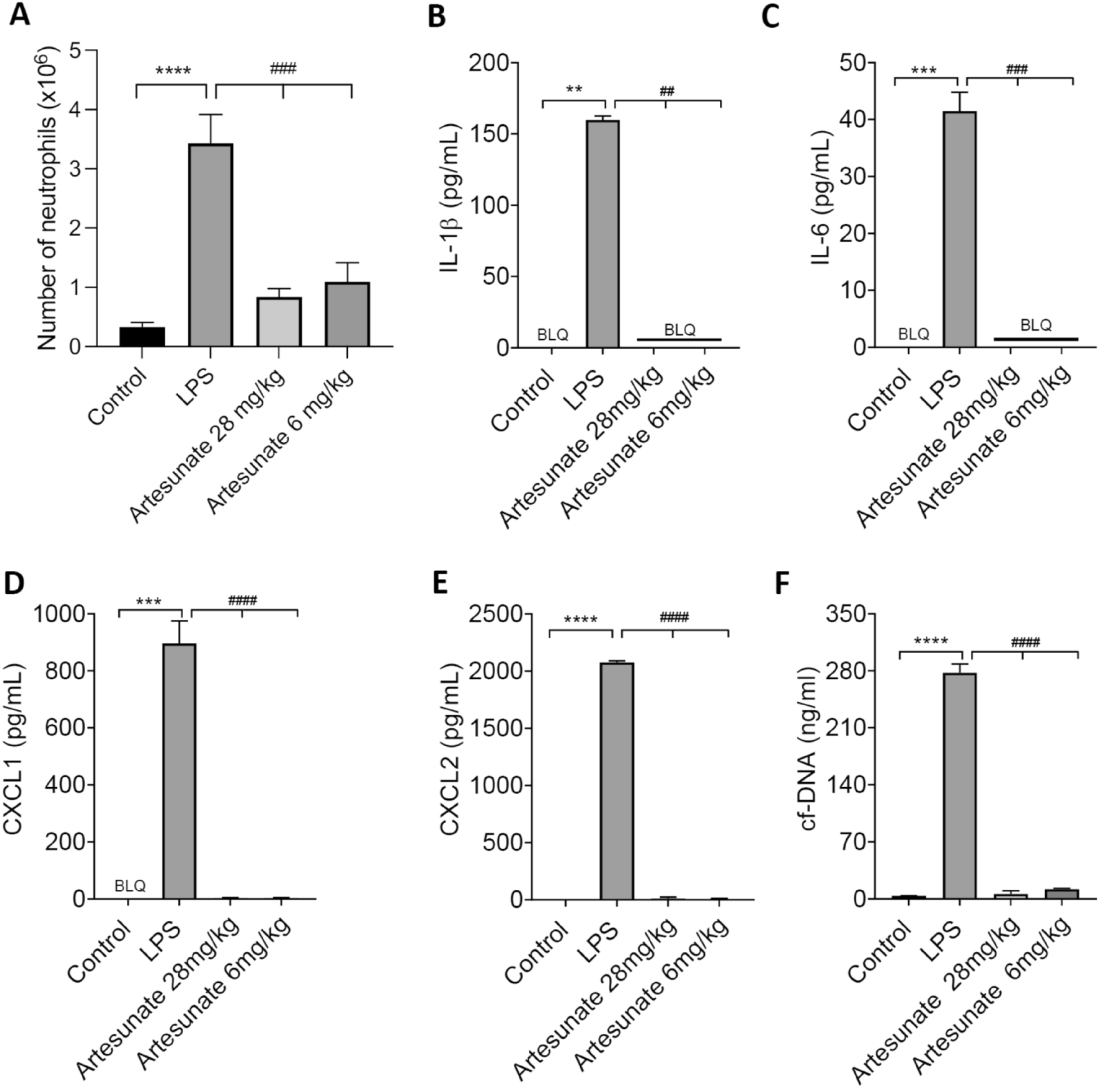
Artesunate suppresses neutrophil invasion, release of cytokines and release of NETs in response to infusion of LPS into lung. (**A**) Number of neutrophils recovered from broncho-alveolar lavage fluid (BALF), 5 h after infusion of LPS (300 ng each lung). LPS causes an influx of neutrophils that is suppressed by artesunate, delivered s.c. at 30 min before and at 90 and 210 min after injection of LPS. Each bar shows mean ± SEM from n = 4 mice. (**B**–**F**) Concentrations of IL-1β, IL-6, CXCL1, CXCL2 and NETs in BALF measured by ELISA and Pico-Green quantification as in Fig. 3, using same experimental protocol as in A. Increase in cytokine concentration and NET release induced by LPS was suppressed by artesunate. Each bar shows mean ± SEM from n = 3 mice. Statistical analysis: BLQ, below limit of quantitation; *p < 0.05; **P < 0.01; ***p < 0.001, ****p < 0.0001, LPS group compared with control group; #p < 0.05; ##p < 0.01; ###p < 0.001; ####p < 0.0001, LPS group compared with LPS plus artesunate group at same time point. ANOVA with Bonferroni post-hoc correction.

The invasion of neutrophils into the lung has been proposed to be critical for the pathogenesis of Covid-19^1–3,5^. We therefore tested whether artemisinin and its analogues are effective in suppressing neutrophil invasion into the lung, and what effect these treatments have on cytokine/chemokine and NET release. Figure 4A shows that lung neutrophil invasion in response to LPS was strongly suppressed by artesunate at both 28 mg/kg and 6 mg/kg, the latter dose being close to the clinically used dose of 2.4 mg/kg. Production of the pro-inflammatory cytokines IL1-β and IL-6, and chemokines CXCL1 and CXCL2, was strongly suppressed (Fig. 4B–E). In addition the release of NETs, as assayed from DNA release, was also inhibited (Fig. 4F).

**Figure 5:**
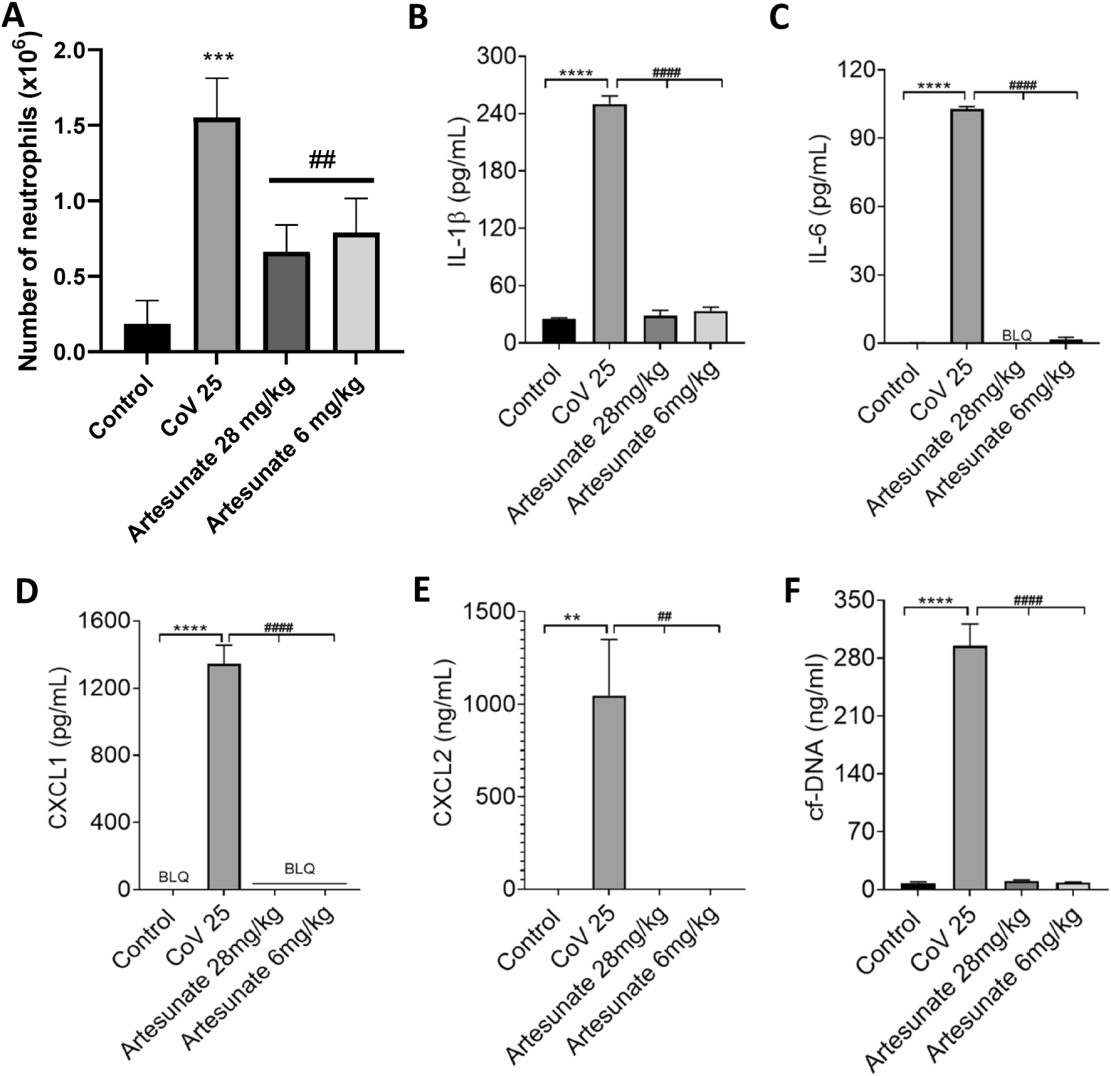
Artesunate suppresses neutrophil invasion, release of cytokines and release of NETs in response to infusion of SARS-CoV-2 spike protein into lung. Number of neutrophils (A), levels of cytokines (B- E) and NETs (F) recovered from broncho-alveolar lavage fluid (BALF), 24 h after infusion of CoV (SARS-CoV-2 spike protein, 25 μg each lung). CoV causes an influx of neutrophils that is suppressed by artesunate, delivered s.c. 30 min before and then at 90 and 210, 330, 450, 570, 690, 810, 1200 and 1320 min after injection of LPS. Each bar shows mean ± SEM from n = 3 mice. BLQ, below limit of quantitation. Statistical analysis: ** p < 0.01, *** p < 0.001, **** p < 0.0001 compared to control; ## p < 0.01, #### p < 0.0001 compared to CoV. (One-way ANOVA and Bonferroni post-hoc test).

A similar experiment was conducted using the SARS-CoV-2 spike protein as chemoattractant (Fig. 5). We found that the peak of neutrophil invasion in response to the SARS-CoV-2 spike protein was delayed compared to LPS, so we assayed neutrophil invasion and the release of cyto/chemokines and NETs at 24 h and maintained levels of artesunate throughout this period by regular injections (see legend to Fig. 5). As was seen with LPS injection, artesunate reduced the invasion of neutrophils into the lungs and also almost totally abolished the release of pro-inflammatory cyto/chemokines and NETs. The dose of 6 mg/kg, close to the dose of 2.4 mg/kg used clinically for malaria, gave approximately the same level of suppression as a higher dose of 28 mg/kg, suggesting that the clinical dose regime used for malaria would also be adequate for treating conditions such as ARDS and Covid-19.

In summary, these experiments show that artemisinin and its analogues potently suppress neutrophil invasion into both peritoneum and lung in response to a wide range of pathological stimuli, and also almost totally inhibit release of cytokines, chemokines and NETs, suggesting that artemisinin may be useful therapeutically in treating conditions such as ARDS and Covid-19 in which cyto/chemokine and NET release are important contributors to morbidity.

### Artemisinin directly suppresses release of cytokines, chemokines and NETs

**Figure 6:**
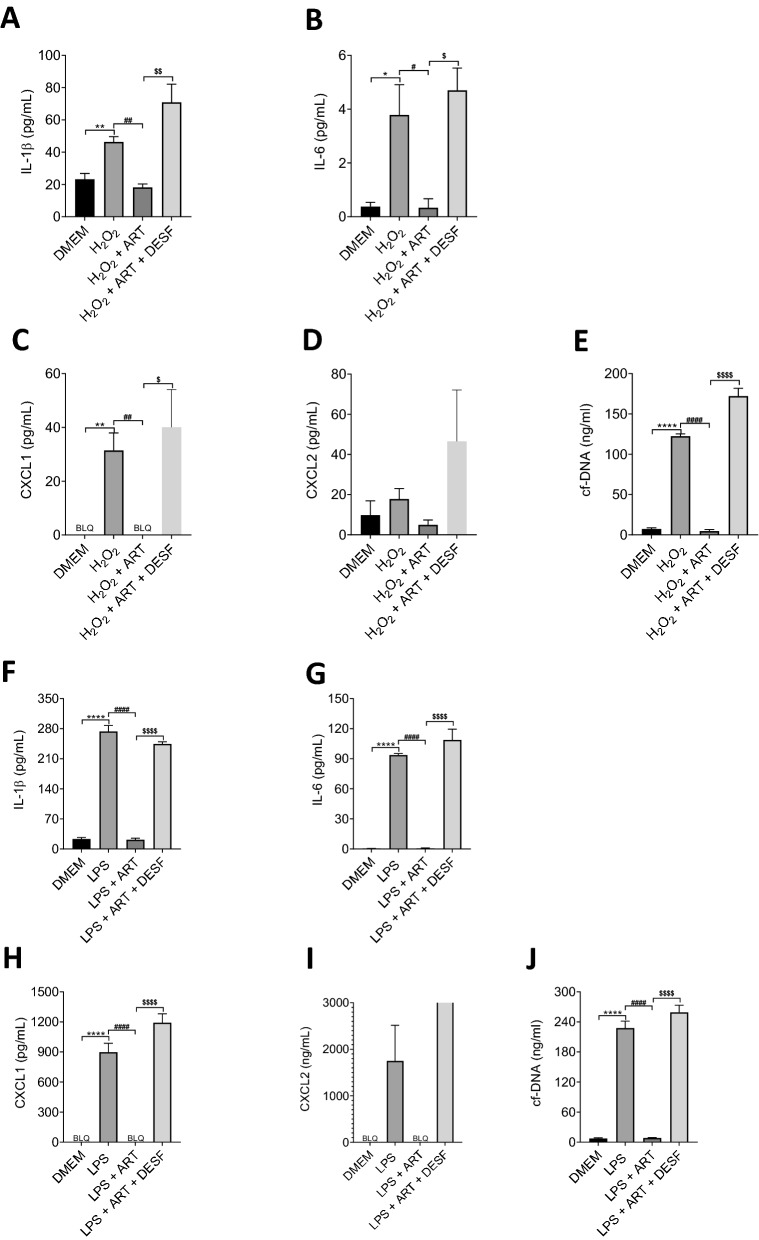
Artesunate directly suppresses cytokine and NET release in response to H_2_O_2_ or LPS in vitro. Graphs show release of cyto/chemokines and NETs from isolated neutrophils after 4 h in culture. Black bar in all graphs represent the release from neutrophils in culture incubated with DMEM only. All other additions were made before the start of the incubation period, as follows: (**A**–**E**), bars show (from left): 10 μM H_2_O_2_; 10 μM H_2_O_2_ plus 10 μM artemisinin; 10 μM H_2_O_2_ plus 10 μM artemisinin plus 50 μM desferioxamine; (**F**–**J**) LPS (10 ng/l); LPS plus artemisinin (10 μM); LPS plus 10 μM artemisinin plus 50 μM desferioxamine. BLQ, below limit of quantitation. Statistical analysis: *p < 0.05; **p < 0.01. ***p < 0.001, ****p < 0.0001 for comparison of H_2_O_2_ or LPS with DMEM control; #p < 0.05; ##p < 0.01. ###p < 0.001, ####p < 0.0001 for comparison of H_2_O_2_ or LPS with H_2_O_2_ /LPS + Artemisinin; and $p < 0.05; $$p < 0.01. $$$p < 0.001, $$$$p < 0.0001 for comparison of H_2_O_2_ or LPS + Artemisinin with H_2_O_2_ or LPS + Artemisinin + Desferrioxamine (One-way ANOVA and Bonferroni post-hoc test).

A notable feature of the data presented in Figs. 3–5 and Supplementary Figs. 10 and 11 is that the inhibition by artemisinin of cyto/chemokine and NET release is in every case greater than the inhibition of neutrophil entry, suggesting that artemisinin may have a dual action: to suppress neutrophil chemotaxis, and in addition to directly suppress release of cyto/chemokines and NETs. In the experiment shown in Fig. 6 we examined the action of the chemoattractants H_2_O_2_ and LPS on isolated neutrophils in order to investigate the possibility of a direct action of artemisinin, independent of inhibition of neutrophil chemotaxis.

A concentration of 10 μM H_2_O_2_, which maximally activates chemotaxis^8^, caused a small but significant enhancement of release of IL-1β, IL-6, CXCL1 and NETs (Fig. 6A–E). The enhancement caused by LPS (10 ng/ml), however, was in each case 1–2 orders of magnitude greater (Fig. 6F–J). In each case the enhanced release caused by both H_2_O_2_ and LPS was completely suppressed by artemisinin, and the action of artemisinin was in turn completely antagonised by the ferrous iron chelator desferrioxamine. These experiments highlight a second action of artemisinin, distinct from its action of inhibiting neutrophil chemotaxis, in directly suppressing release of cyto/chemokines and NETs. The mechanism of this action is currently unknown but appears to be distinct from the action on chemotaxis, suggesting the existence of a second target of artemisinin that controls the release of inflammatory mediators from neutrophils. A second target for artemisinin would not be surprising, as previous studies have also shown artemisinin to have broad effects on a number of systems in malarial parasites, including glycolytic pathways, haemoglobin degradation, antioxidant defence and protein synthesis^31,32^.

## Discussion

The work described here shows that artemisinin and its active analogues are potent inhibitors of mammalian neutrophil and macrophage chemotaxis. We find that artemisinin inhibits chemotaxis by blocking the generation of leading-edge calcium signals that are required for innate immune cell chemotaxis. The target of artemisinin in inhibiting chemotaxis is the SERCA3 calcium pump isoform that is responsible for filling neutrophil intracellular stores with calcium, with the effect that intracellular stores are emptied and leading-edge calcium signals can therefore no longer be generated. Artemisinin inhibits only one isoform, SERCA3, out of the three mammalian SERCA isoforms, a selectivity that explains the lack of toxicity of artemisinin when used clinically as an antimalarial. We also find that artemisinin and its analogues are highly effective at reducing neutrophil chemotaxis and inhibiting cytokine/chemokine and NET release both in vivo and in vitro, and in both peritoneum and lung.

There are several reasons for thinking that the mechanism of action of artemisinin is the same for inhibition of neutrophil chemotaxis and killing of malaria parasites. In both cases the potency is high (IC_50_ ≈ 5 nM for malarial killing^33^ vs. IC_50_ ≈ 0.3 nM for inhibition of neutrophil chemotaxis, see Fig. 1); efficacy is completely abolished in both cases by replacing the unusual peroxide bridge with a single oxygen (ref.^18^ and Fig. 2A); and the action depends in both cases on low micromolar concentrations of free ferrous iron as a catalyst (refs^18,34^ and Fig. 1E,F). Thus, discovering the mechanism of action of artemisinin in inhibiting neutrophil chemotaxis is likely to give clues to the mechanism of action in killing malaria parasites. Understanding the molecular basis of the anti-malarial action of artemisinin will open up the possibility of designing novel antimalarials based on the artemisinin scaffold, which may become essential in the face of growing malarial resistance to artemisinin and its analogues. Previous work has identified multiple targets of artemisinin in the malaria parasite that are covalently modified by artemisinin^31,32^, but in these studies the artemisinin-derived probes were used at a concentration three orders of magnitude or more above the IC_50_ value of 0.3 nM for mammalian SERCA3 found in the present study, so the possibility of a more selective effect at lower concentrations of artemisinin cannot be excluded.

The lack of toxicity of artemisinin in mammals, which express three SERCA isoforms, is explained because the critical isoforms SERCA1 and 2 are insensitive to artemisinin (Fig. 2E–G). Malaria parasites, on the other hand, express a single SERCA isoform (also known as PfATP6)^18^. Malarial SERCA was proposed some years ago to be the target of artemisinin^18^, but subsequent studies did not confirm this work^35,36^ and the idea has remained controversial in the field. The work in the present paper suggests that malarial SERCA is indeed likely to be the target of artemisinin, as was originally proposed^18^.

How can artemisinin achieve selective inhibition of SERCA3 but not the closely-related isoforms SERCA1 and SERCA2? Alkylation of a specific cysteine residue in SERCA3 could be achieved if a high-affinity binding pocket for artemisinin was located adjacent to the target cysteine residue in SERCA3 but not in other isoforms. The SERCA pump undergoes large structural rearrangements during its active cycle^37^, and it is therefore plausible that the addition of a bulky residue such as artemisinin, coupled irreversibly to a cysteine residue in a critical location, could be responsible for inhibiting the calcium transporter function.

Here we also show that artemisinin and its analogues are potent inhibitors of neutrophil invasion into peritoneum and lung in vivo in response to chemoattractants such as H_2_O_2_, LPS and the SARS-CoV-2 spike protein from the virus that causes Covid-19. The knowledge that an important target of artemisinin is SERCA3 gives a molecular basis for past empirical studies using artemisinin in rodent models of lung inflammation and sepsis *in vivo*^38–45^. These studies have shown that artemisinin and its analogues inhibit cytokine release, reduce lung pathology and significantly enhance survival in response to insults such as lung infusion of lipopolysaccharide or bleomycin, exposure to cigarette smoke, and inflammation caused by systemic sepsis, and moreover that artemisinin appeared to have no adverse effects, even at large doses^38–45^. Our results complement these studies by showing that artemisinin and its analogues inhibit cytokine/chemokine release following injection of both LPS, a bacterial cell wall component, and the SARS-CoV-2 spike protein. Moreover, they also show a striking effect in inhibiting NET release.

Is inhibition of neutrophil chemotaxis the only mechanism by which artemisinin blocks the release of cyto/chemokines and NETs? While simply preventing the entry of neutrophils into organs such as lung or peritoneum undoubtedly makes an important contribution to inhibiting the release of pro-inflammatory factors such as cytokines and NETs in vivo, the work shown here suggests that a more direct inhibition also makes an important contribution, for two reasons: the inhibition of neutrophil chemotaxis in vivo is less complete than the inhibition of release of pro-inflammatory factors; and artemisinin has a potent effect on release of pro-inflammatory factors in vitro. An important second target of artemisinin, whose inhibition blocks synthesis or release of proinflammatory factors, therefore remains to be discovered.

Together with previous work, the results presented here suggest that artemisinin may have value in enhancing survival in conditions such as sepsis, ARDS and Covid-19. Much of the work presented in this paper formed the basis of a proposal to the World Health Organisation (WHO) for the use of artesunate as a therapy for patients seriously ill with Covid-19. This idea is now in clinical trials as part of the ‘SOLIDARITY’ initiative^46–48^.

## Materials and methods

### Animals

Black C57BL/6 WT mice (6–8 weeks old) were purchased from Charles River Inc. All animal work was conducted under UK Home Office personal and project licences, approved by the Animal Welfare Ethical Review Board (AWERB) of King’s College London and carried out in accordance with the Animals (Scientific Procedures) Act 1986 and in compliance with the ARRIVE guidelines.

### Chemicals and reagents

Hydrogen peroxide (H_2_O_2_, 31642), adenosine 5′diphosphoribose (ADPR, A0752), thioglycolate (70157), lipopolysaccharide (LPS, LPS25), CXCL2 (SRP4251), complement component 5a (C5a, SRP4895A), *N*-(p-Amylcinnamoyl)anthranilic acid (ACA, A8486), lumefantrine (PHR2186), mefluoquine (PHR1705), hydroxychloroquine (PHR1782) and arteether (SML2592) were purchased from Sigma-Aldrich (Sigma-Aldrich Company Ltd., Dorset, UK). Pluronic F-127 (P3000MP), Fura-2AM (F1221) and Sytox Green (S7020) were purchased from Thermo Fisher Scientific (Thermo Fisher Scientific Life Technologies, Waltham, Massachusetts, U.S). BAPTA-AM was purchased from Stratech Scientific Ltd. The RAL DIFF-QUIK kit (a modified version of the May-Grünwald-Giemsa stain) (RAL555) was purchased from RAL Diagnostics, (RAL Diagnostics, Martillac, France). Deoxyartemisinin (2-deoxy-artemisinin) (20428) and artemether (11815) were purchased from Cambridge Bioscience (Cambridge UK). Artesunate (A3731), thapsigargin (T9033), desferrioxamine (BP987), L-cysteine (168149), pyrimethamine (BP1227) and dihydroartemsinin (1200520) were purchased from Merck Life Sciences (Feltham, UK). The XTT cell viability kit (9095S) was purchased from New England Biolabs (Ipswich, Massachusetts, U.S.) SARS-CoV-2 spike protein was sourced from R&D Systems, Bio-Techne. The natural products: artemisinin, beta-carotene, bisabolol, capsaicin, carvacrol, citral, citronellal, curcumin, D-biotin, ergosterol, eugenol, farnesene, farnesol, ferulic acid, gallic acid, geraniol, hesperidin, isoeugenol, lanosterol, lawsone, limonene, myrcene, *N*-acetylcysteine, neomenthol, (+)—pulegone, (−)—pulegone, quercetin, rutin-hydrate, thymol, vanillin and veratrylamine were kindly donated by Dr Suaib Luqman from CSIR-Central Institute of Medicinal and Aromatic Plants, Lucknow-226015, Uttar Pradesh, India. Artemisinin and analogues, some of which have limited solubility, were dissolved at 10 mM in 90% DMSO/10% TWEEN to make stock solutions that were then diluted as appropriate on the day of the experiment.

### Isolation of mouse peritoneal neutrophils and macrophages

#### In vitro* chemotaxis experiments*

Mice were injected i.p. with 3% thioglycolate solution (10 μl/g) and, after 4 h (for neutrophils) or 4 d (for macrophages), were euthanised by cervical dislocation. The peritoneal-covering skin was removed, 5 ml PBS injected into the peritoneal cavity which was massaged gently for 60 s to dislodge cells. The peritoneal fluid was gently extracted by syringe and centrifuged for 10 min at 200 RCF. The supernatant was discarded and cells resuspended in DMEM + 10% FBS. These methods generated cell suspensions containing > 90% of either neutrophils or macrophages, identified through a fast-acting modified version of the May-Grünwald-Giemsa staining and subsequent cell type identification as shown in^8^ (neutrophils) and Supplementary Fig. 3 (macrophages).

#### In vivo* peritoneal chemotaxis experiments*

Mice were injected i.p. with H_2_O_2_ or LPS and at experimental time points (see methods below), mice were euthanised by cervical dislocation. The peritoneal lavage was recovered as above, and samples of the suspensions were immediately spun down onto glass slides using a cytocentrifuge (Sigma 2–7 Cyto, Shandon, Germany as described below) and leukocytes (neutrophils, macrophages) identified through a fast-acting modified version of the May-Grünwald-Giemsa staining and subsequent cell type identification as shown in^8^. The remaining cell suspension was then centrifuged for 10 min at 200 RCF and supernatants were collected and frozen at − 20 °C for cyto/chemokine analysis by ELISA and cf-DNA(NET) quantification using Quant-iT PicoGreen kit (Thermo Fisher).

### Isolation of mouse BALF neutrophils

The nostrils of mice briefly anaesthetized were infused with H_2_O_2_, LPS or SARS-CoV-2 spike protein and at experimental time points (see lung methods below), mice were euthanised by destruction of the brain. The mice were placed in the supine position, limbs were secured and the skin around the neck was removed. Salivary glands were separated to reveal the sternal hyoid muscle and forceps used to incise the muscle around the trachea. A cotton suture was then threaded under the tracheal tissue. A needle was then used to puncture the middle of the trachea between two cartilage rings and a pre-made plastic catheter was inserted ~ 0.5 cm into the tracheal lumen and stabilised with the suture. A syringe, loaded with 1 ml PBS was then attached to the catheter and PBS slowly injected. The thorax was massaged gently for 60 s, before BAL fluid was aspirated. This was repeated 3 times to maximise the BAL fluid recovery.

Samples of the BAL fluid were immediately spun down onto glass slides using a cytocentrifuge (as described below) and neutrophils identified through a fast-acting modified version of the May-Grünwald-Giemsa staining and subsequent cell type identification as shown in ^8^. The remaining cell suspensions was then centrifuged for 10 min at 200 RCF and supernatants were collected and frozen at − 20 °C for cyto/chemokine analysis by ELISA and cf-DNA (NET) quantification using Quant-iT PicoGreen kit (Thermo Fisher).

### Cell identification in peritoneal and BALF extracts

Cell suspension was isolated from peritonea/lungs of WT mice as above, spun down onto glass slides using a cytocentrifuge at 400 RPM for 5 min and left to air-dry overnight. A modified version of the May-Grünwald-Giemsa staining was used to identify cell types (RAL DIFF-QUIK kit, RAL diagnostics). Slides were suspended in RAL Diff-Quick fixative solution (methanol based solution to stabilize cellular components) for 1 min, in RAL Diff-Quik solution I (Xanthene solution; a buffered solution of Eosin Y) for 1 min and in RAL Diff-Quik solution II (a buffered solution of thiazine dyes, consisting of methylene blue and Azure A) for 1 min. Nuclei were meta-chromatically stained red/purple and cytoplasm pink/yellow (see ref^8^ and Supplementary Fig. 4).

### Neutrophil and macrophage chemotaxis assays

Ibidi µ-slide chemotaxis assay chambers, precoated with collagen IV along the central migration strip, were purchased from Thistle Scientific Ltd (Uddingston, Glasgow, UK). Neutrophils or macrophages, isolated as above from peritonea of WT mice, were re-suspended within 30 min of collection in DMEM + 10% FBS at a concentration of 5 × 10^5^ cells per ml and 6 µl was seeded along the central migration strip of an Ibidi µ-slide chamber as per the manufacturer’s instructions. Slides were incubated for 1 h at 37 °C in humidified 95% air/5% CO_2_, to allow neutrophil/macrophage adherence to the central migration strip. DMEM (without added FBS) with and without added chemoattractant was then added to the wells on opposite sides of the central migration strip. DMEM was from Thermo Fisher Scientific Cat. No. 41966-029. For experiments in which effects of compounds were to be tested, equal concentrations were added to both DMEM + chemoattractant and DMEM wells. Slides were pre-incubated at 37 °C in 95% air/5% CO_2_ for 20 min to allow the generation of a gradient of chemoattractant across the 1 mm wide × 70 μm deep central cell migration strip. Live-cell time-lapse microscopy was then conducted using a 10 × lens and dark-field illumination on a Nikon Eclipse Ti-E inverted microscope equipped with the Nikon Perfect Focus System (PFS). The microscope was housed in a temperature-controlled Perspex box (Solent Scientific) at 37 °C, with slides housed in a stage-mounted block in humidified 95% air/5% CO_2_. A maximum of 12 individual chambers (4 individual slides, 3 chambers per slide) could be imaged per experiment by using a motorized stage. Stage movement, lens focus and image acquisition were controlled by Nikon NIS Elements software. Experiments were conducted over 2 h for neutrophils and 1 h for macrophages, with images of each assay compartment taken every 2 min. The ImageJ Fiji TrackMate plug-in was employed to track individual neutrophils/macrophages. A chemotaxis and migration plug-in, provided by Ibidi, was used to calculate speed and forwardl migration index (FMI) data from the neutrophil/macrophage tracks. For further details see ref ^8^.

### Calcium imaging of neutrophils

Neutrophils isolated as above from the peritonea of WT mice, were re-suspended in DMEM + 10% FBS at a concentration of 5 × 10^5^ per ml. Neutrophils were plated onto a collagen-coated 13 mm round glass coverslip and incubated at 37 °C in 95%air/5% CO_2_ for 1 h to allow neutrophils to adhere. Fura2-AM (5 µM in DMEM) was then added to the cells on the coverslip for 30 min at 37 °C in 95% air/5% CO_2_. Solutions were changed as shown in the figures and fluorescence was measured during alternating illumination at 340 nm and 380 nm (OptoScan; Cairn Research Inc, Kent, UK) every 2 s using a Nikon Eclipse Ti inverted microscope with a 40 × lens and iXon 897 EM-CCD camera controlled by WinFluor 3.2 software. F_340/380_ ratios were obtained using FIJI (ImageJ) and converted to calcium concentrations using the equation given by Grynkiewicz et al. with values R_max_ = 2.501, R_min_ = 0.103, both determined experimentally*.*

For experiments when calcium signals during chemotaxis up a gradient of chemoattractant were to be recorded (as in Supplementary Video 1), 1 µl of Fura-2 AM solution (50 µg Fura-2 AM + 10 µl pluronic F-127 + 10 µl DMSO) was added to 500 µl of peritoneal neutrophil suspension and incubated for 1 h at 37 °C in 95%air/5% CO_2_. Fura-2 loaded cells in suspension were seeded into Ibidi chambers as described above and imaged in a Nikon Ti-E microscope with a 40 × phase contrast lens. Fast-moving neutrophils located in the middle of the central cell migration strip were selected, with typically only one cell imaged per field. Calcium ratio images were obtained with alternating 340 nm and 380 nm epi-illumination supplied by stable LED light sources (Fura-LED, Cairn Research), at 500 ms intervals. All images were filtered by a broad-band 510 nm filter and captured with a Photometrics Prime 95B sCMOS camera. Stage movement, focus and image acquisition were controlled by Nikon NIS Elements software. The ImageJ Fiji RatioPlus plug-in was used to generate F_340/380_ ratio images and a rainbow look-up table (LUT) was applied to the ratio images to indicate the level of calcium. For further details see ref^8^.

### Loading cells with BAPTA-AM

To determine the effect of intracellular calcium chelation on intracellular calcium levels and chemotaxis induced by H_2_O_2_, extracted mouse peritoneal neutrophils were re-suspended in DMEM + 10% FBS at a concentration of 5 × 10^5^ per ml and for chemotaxis experiments were incubated with or without BAPTA-AM (50 µmol/l, Stratech Scientific Ltd) for 30 min. To measure the effect of BAPTA on intracellular calcium levels neutrophils were also incubated with Fura2-AM as described above.

### Transfection of HEK293 cells

Human embryonic kidney HEK293 cells were split at a confluency of 80%, resuspended in media to a concentration of 7 × 10^4^ cells per ml and 0.5 ml was plated into a four-well plate containing 13 mm glass coverslips pre-coated with poly-d-lysine (1 mg/ml), ready for transfection the following day. Cells were transfected with 0.5 µg of a plasmid containing cDNA for SERCA1, 2 or 3 using a modified calcium-phosphate protocol, as previously described^49^. Cells were used for calcium imaging 2d post-transfection.

Rat SERCA1a (pMT2) was a gift from Jonathan Lytton (Addgene plasmid # 75182; http://n2t.net/addgene:75182; RRID: Addgene_75182)^50^. Human SERCA2a (pcDNA3.1+) was a gift from Jonathan Lytton & David MacLennan (Addgene plasmid # 75187; http://n2t.net/addgene:75187; RRID: Addgene_75187)^51^. Human SERCA3 (pMT2) was a gift from Jonathan Lytton & David MacLennan (Addgene plasmid # 75189; http://n2t.net/addgene:75189; RRID: Addgene_75189)^52^.

### Patch clamp

Transfection of HEK293 cells with TRPM2, a kind gift from Prof Y. Mori, University of Kyoto, Japan, was carried out as described above. Manual whole-cell patch clamp recording was carried out as previously described^53^. TRPM2 ion channels were activated by the inclusion of 1 mM ADPR in the intracellular patch clamp solution.

### XTT cell viability assay

Peritoneal neutrophils, isolated as above, were seeded into four individual 96 well plates (2 × 10^5^/well) and incubated for 1 h at 37 °C in 95% air/5% CO_2_ to allow adherence. Artemisinin was then added to half of the wells on all plates at a 10 µM concentration. Following incubation for: 0 h, 12 h, 24 h and 48 h, respectively, 50 µL of XTT/PMS solution was added to all wells, and plates were incubated for a further 2 h, before absorbance was analysed on a FLUOstar Omega microplate reader (BMG LABTECH, Buckinghamshire, UK) at 450 nm.

### In vivo peritoneal H_2_O_2_ chemotaxis experiments

WT mice were injected s.c. with either sham or artemisinin/artesunate (either 28 mg/kg or 6 mg/kg for both) 30 min prior to being injected i.p. with H_2_O_2_ (10 µM in PBS, 10 µl/g body weight) or PBS alone for the control baseline group. Mice were then euthanised over 10–210 min and peritoneal lavage was extracted and cell types identified as described above, before supernatants were analysed for cytokines/chemokines by ELISA and for NETs by cf-DNA quantification.

### In vivo peritoneal LPS chemotaxis experiments

WT mice were injected s.c. with either sham or artemisinin (28 mg/kg) 30 min prior to being injected i.p. with LPS (30 ng/cavity) or PBS alone for control group. Further sham/artemisinin s.c. injections were administered at 90 and 210 min, before mice were euthanised at 300 min and peritoneal lavage was extracted and cell types identified as described above, before supernatants were analysed for cytokines/chemokines by ELISA and for NETs by cf-DNA quantification.

### Lung BALF H_2_O_2_ chemotaxis experiments

WT mice were injected s.c. with either sham or artesunate (28 mg/kg or 6 mg/kg) 30 min prior to having H_2_O_2_ (10 µM in PBS) or PBS alone for control group infused into both nostrils. Mice were euthanised after 60 min and bronchio-alveolar lavage fluid (BALF) was extracted and cell types identified as described above, before supernatants were analysed were analysed for cytokines/chemokines by ELISA and for NETs by cf-DNA quantification (see below).

### Lung BALF LPS and SARS-CoV-2 spike protein chemotaxis experiments

WT mice were injected s.c. with either sham or artesunate (28 mg/kg or 6 mg/kg) 30 min prior to having LPS (300 ng in PBS each lung), SARS-CoV-2 spike protein (25 μg in PBS each lung) or PBS alone for the control group infused into both nostrils. Further sham/artesunate s.c. injections were administered at 90 and 210 min, before mice were euthanised at 300 min and BALF lavage was extracted and cell types identified as described above, before supernatants were analysed were analysed for cytokines/chemokines by ELISA and for NETs by cf-DNA quantification.

### Analysis of cytokines and chemokines in peritoneal and lung fluid

At the indicated times after injection of the stimuli (H_2_O_2_, LPS or SARS-CoV-2 spike protein), animals were terminally anesthetized and the peritoneal lavage or BALF was collected in PBS. IL-6, IL-1β, CXCL1 and CXCL2 concentrations were measured by enzyme-linked immunosorbent assay (ELISA) using commercial kits (DuoSet; R&D Systems) as previously described^54^. The results are expressed as pg/mL of each cytokine/chemokine. As a control, concentrations of these cytokines/chemokines were measured in mice injected with vehicle (PBS).

### Quantification of cell free DNA (NETs) in peritoneal and lung fluid

Peritoneal lavage or BALF were collected at different time points after injection of stimuli (H_2_O_2_, LPS or SARS-CoV-2 spike protein) and the amount of cell free DNA (cf-DNA) was quantified using the Quant-IT™ PicoGreen® kit (Thermo Fisher) according to the manufacturer’s instructions. The fluorescence intensity (excitation at 488 nm and emission at 525 nm wavelength), a measure of the amount of dye bound to DNA, was quantified by a fluorescence reader (FlexStation 3 Microplate Reader, Molecular Devices, CA, USA) as previously described^55^. The results are expressed as ng/mL of cf-DNA.

### Imaging of NETs

Extracted mouse peritoneal neutrophils were re-suspended in DMEM + 10% FBS at a concentration of 5 × 10^5^ per ml and incubated with or without 10 ng/l LPS (4 h). To examine the effect of artemisinin neutrophils were pre-treated for with 10 µM artemisinin (30 min before LPS incubation). Samples were then incubated for 1 h with Sytox green nucleic acid stain (5 µM) (Thermo Fisher Scientific). Cells were plated onto coverslips and illuminated using 488 nm wavelength light at 10 × or 60 × magnification to visualise release of DNA from the neutrophils as NETs. Cells were classed as having released NETs if the diameter of the fluorescent area was > 2 × that of average for untreated cells.

## Supplementary Information

Supplementary Information 1.

Supplementary Information 2.
