# Supplementary material for: Artemisinin inhibits neutrophil and macrophage chemotaxis, cytokine production and NET release
Source: Sci Rep. 2022 Jun 30;12:11078. doi: 10.1038/s41598-022-15214-6 (PMC9245885; doi:10.1038/s41598-022-15214-6)
Supplement: Supplementary file 1 — Supplementary Information 1. [file 41598_2022_15214_MOESM1_ESM.pptx]

## Slide 1
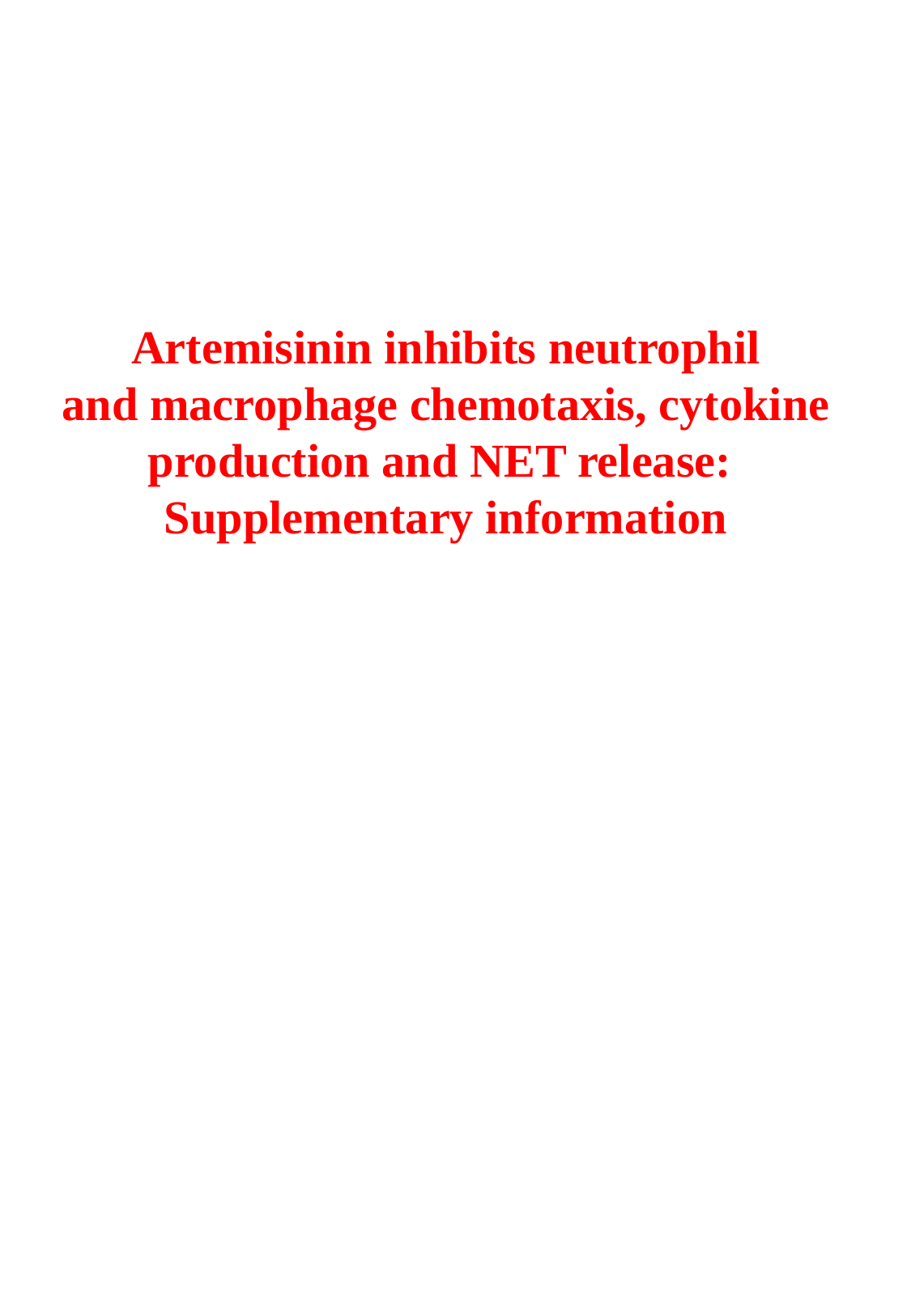

Artemisinin inhibits neutrophil
and macrophage chemotaxis, cytokine production and NET release:
Supplementary information

## Slide 2
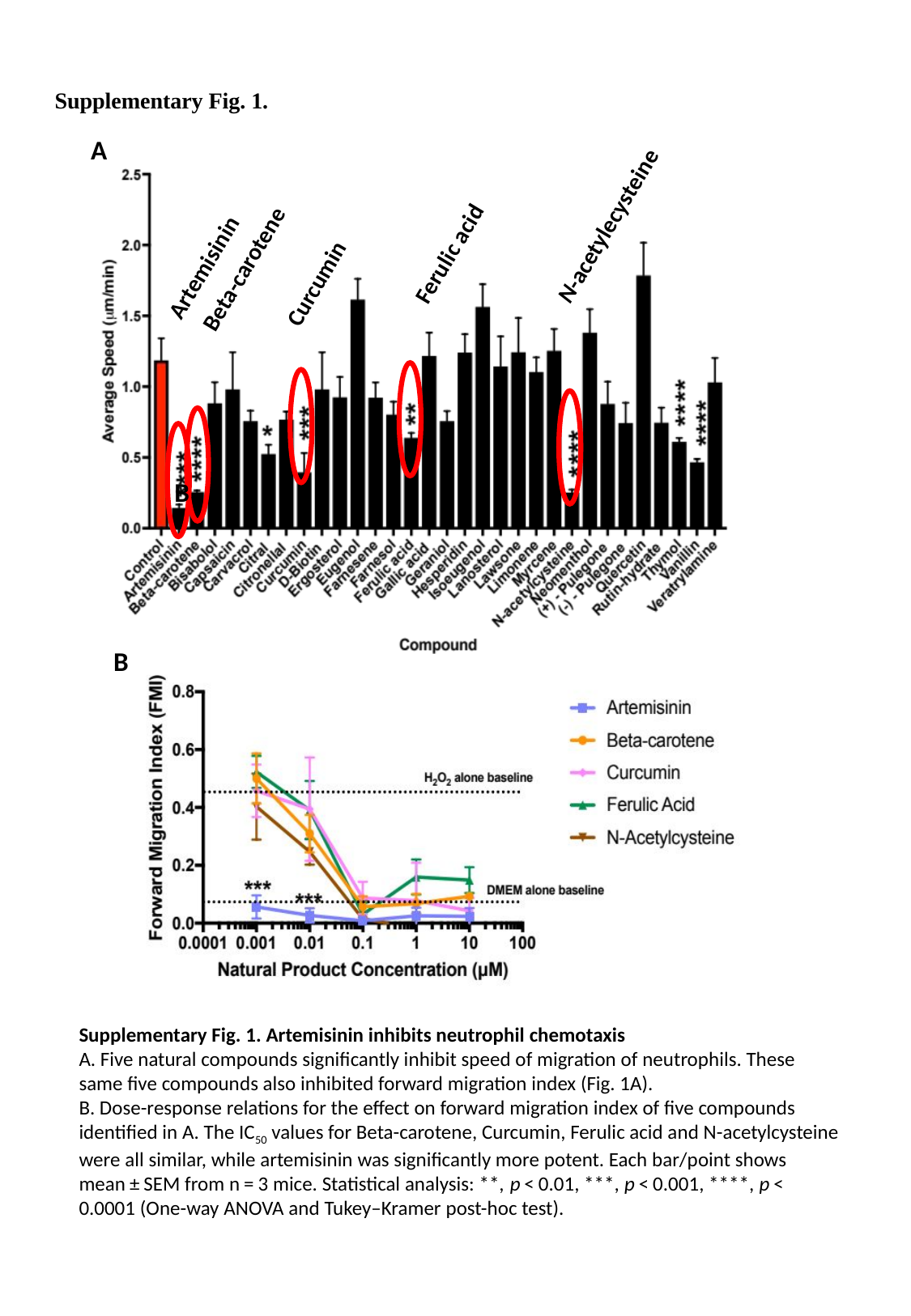

N-acetylecysteine
Ferulic acid
Artemisinin
Curcumin
Beta-carotene
Supplementary Fig. 1.
A
B
B
Supplementary Fig. 1. Artemisinin inhibits neutrophil chemotaxis
A. Five natural compounds significantly inhibit speed of migration of neutrophils. These same five compounds also inhibited forward migration index (Fig. 1A).
B. Dose-response relations for the effect on forward migration index of five compounds identified in A. The IC50 values for Beta-carotene, Curcumin, Ferulic acid and N-acetylcysteine were all similar, while artemisinin was significantly more potent. Each bar/point shows mean ± SEM from n = 3 mice. Statistical analysis: **, p < 0.01, ***, p < 0.001, ****, p < 0.0001 (One-way ANOVA and Tukey–Kramer post-hoc test).

## Slide 3
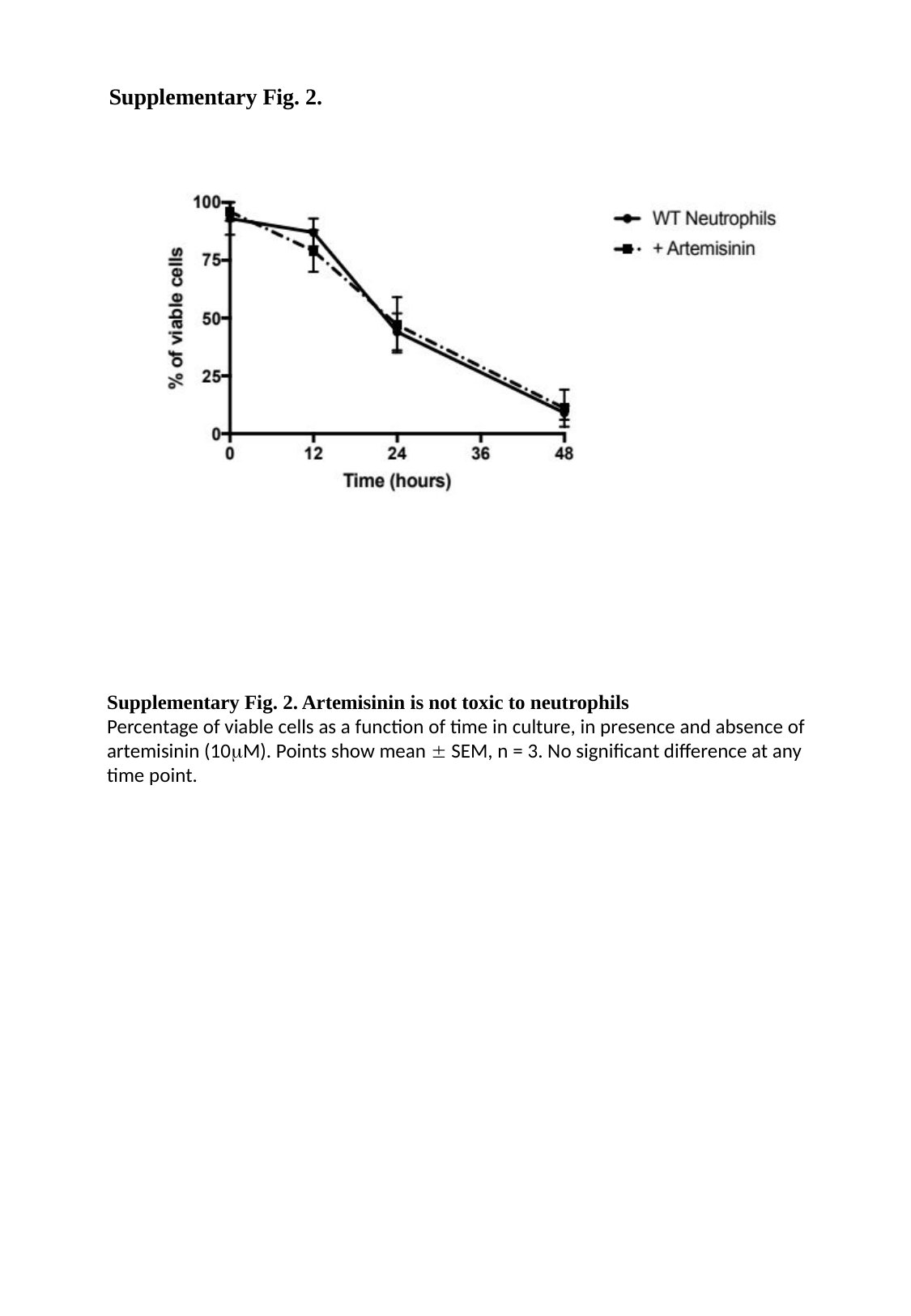

Supplementary Fig. 2.
Supplementary Fig. 2. Artemisinin is not toxic to neutrophils
Percentage of viable cells as a function of time in culture, in presence and absence of artemisinin (10μM). Points show mean ± SEM, n = 3. No significant difference at any time point.

## Slide 4
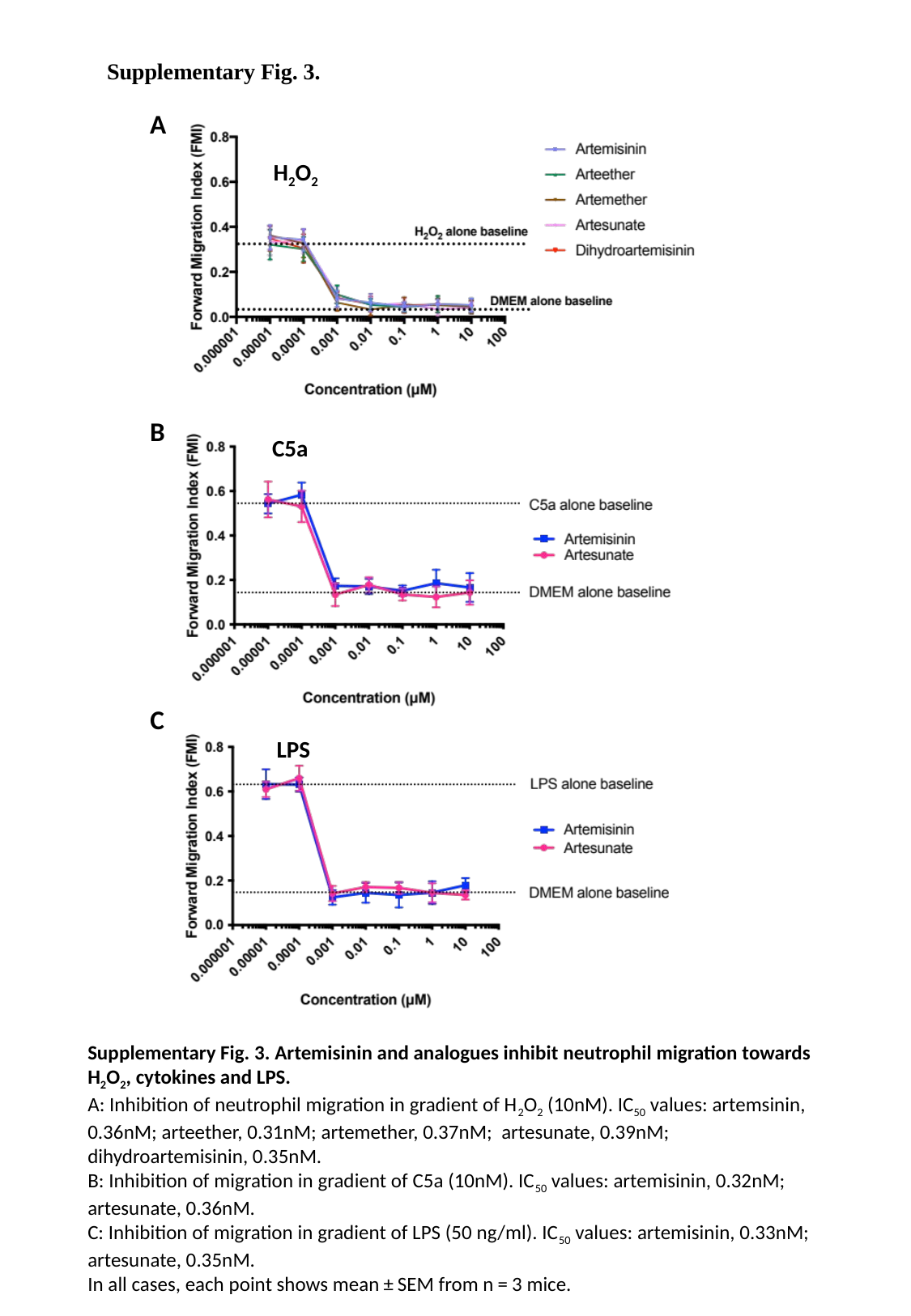

Supplementary Fig. 3.
A
H2O2
B
C5a
C
LPS
Supplementary Fig. 3. Artemisinin and analogues inhibit neutrophil migration towards H2O2, cytokines and LPS.
A: Inhibition of neutrophil migration in gradient of H2O2 (10nM). IC50 values: artemsinin, 0.36nM; arteether, 0.31nM; artemether, 0.37nM; artesunate, 0.39nM; dihydroartemisinin, 0.35nM.
B: Inhibition of migration in gradient of C5a (10nM). IC50 values: artemisinin, 0.32nM; artesunate, 0.36nM.
C: Inhibition of migration in gradient of LPS (50 ng/ml). IC50 values: artemisinin, 0.33nM; artesunate, 0.35nM.
In all cases, each point shows mean ± SEM from n = 3 mice.

## Slide 5
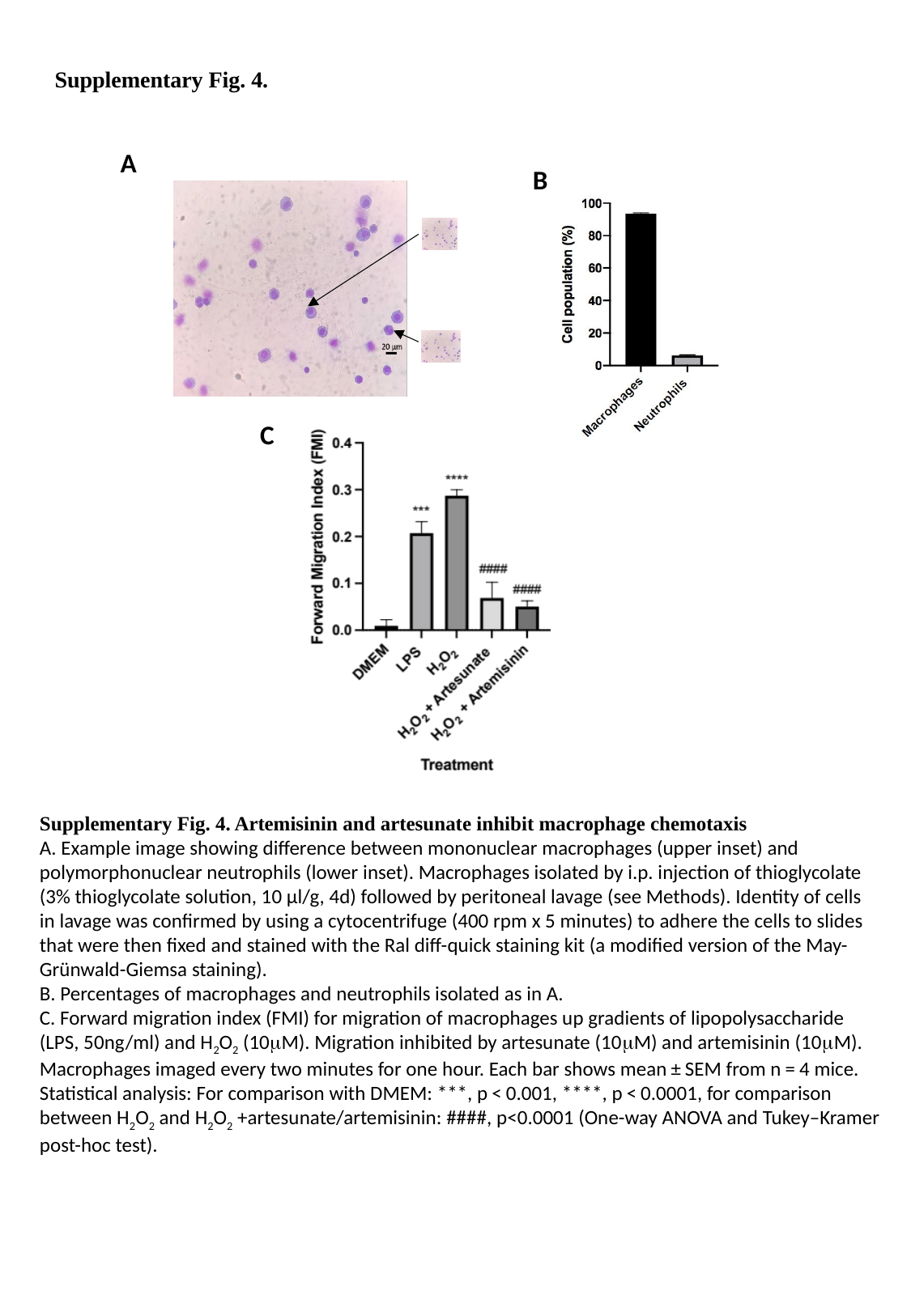

Supplementary Fig. 4.
A
B
C
Supplementary Fig. 4. Artemisinin and artesunate inhibit macrophage chemotaxis
A. Example image showing difference between mononuclear macrophages (upper inset) and polymorphonuclear neutrophils (lower inset). Macrophages isolated by i.p. injection of thioglycolate (3% thioglycolate solution, 10 μl/g, 4d) followed by peritoneal lavage (see Methods). Identity of cells in lavage was confirmed by using a cytocentrifuge (400 rpm x 5 minutes) to adhere the cells to slides that were then fixed and stained with the Ral diff-quick staining kit (a modified version of the May-Grünwald-Giemsa staining).
B. Percentages of macrophages and neutrophils isolated as in A.
C. Forward migration index (FMI) for migration of macrophages up gradients of lipopolysaccharide (LPS, 50ng/ml) and H2O2 (10μM). Migration inhibited by artesunate (10μM) and artemisinin (10μM). Macrophages imaged every two minutes for one hour. Each bar shows mean ± SEM from n = 4 mice. Statistical analysis: For comparison with DMEM: ***, p < 0.001, ****, p < 0.0001, for comparison between H2O2 and H2O2 +artesunate/artemisinin: ####, p<0.0001 (One-way ANOVA and Tukey–Kramer post-hoc test).

## Slide 6
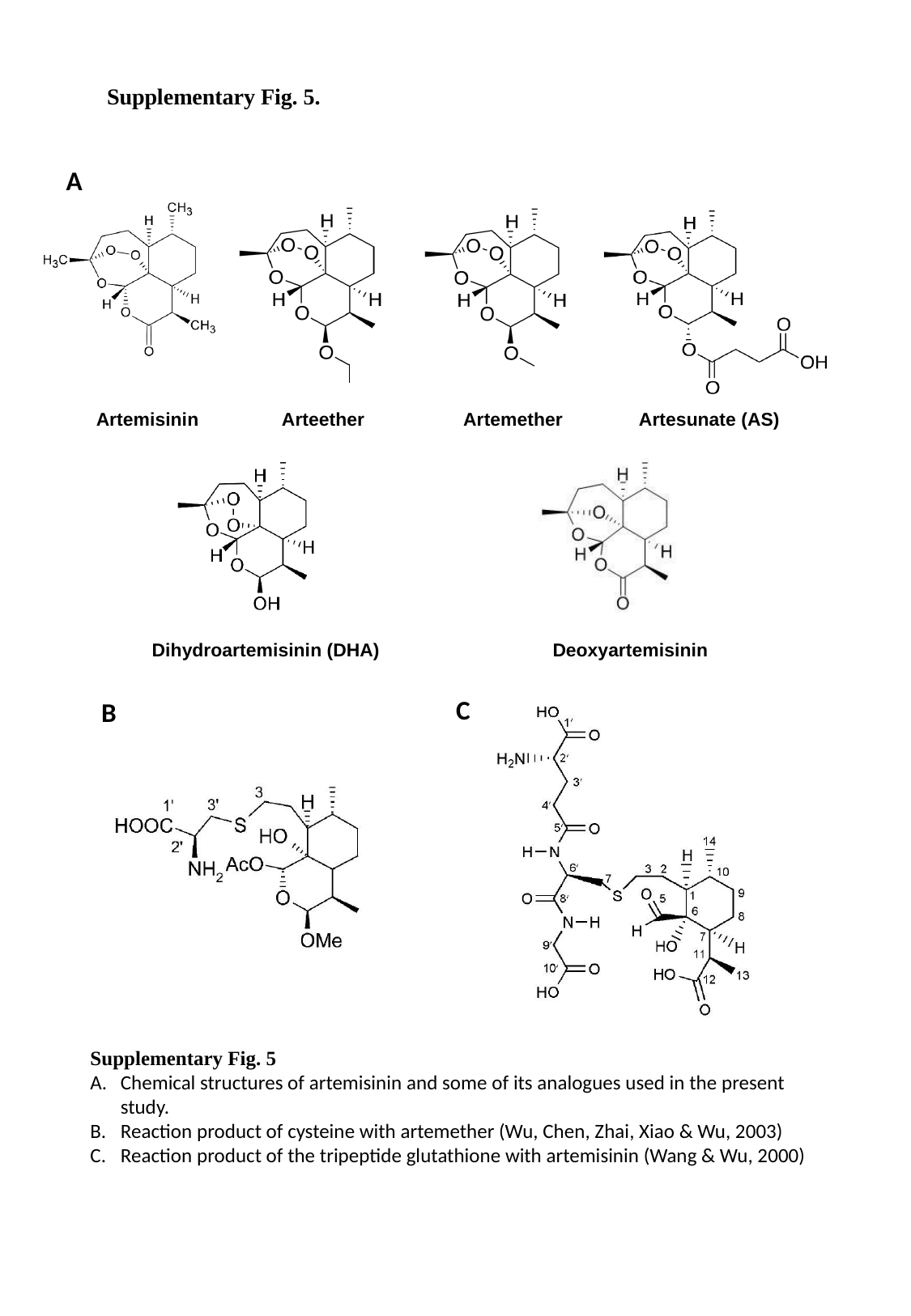

Supplementary Fig. 5.
A
 Artemisinin Arteether Artemether	 Artesunate (AS)
Dihydroartemisinin (DHA) Deoxyartemisinin
C
B
Supplementary Fig. 5
Chemical structures of artemisinin and some of its analogues used in the present study.
Reaction product of cysteine with artemether (Wu, Chen, Zhai, Xiao & Wu, 2003)
Reaction product of the tripeptide glutathione with artemisinin (Wang & Wu, 2000)

## Slide 7
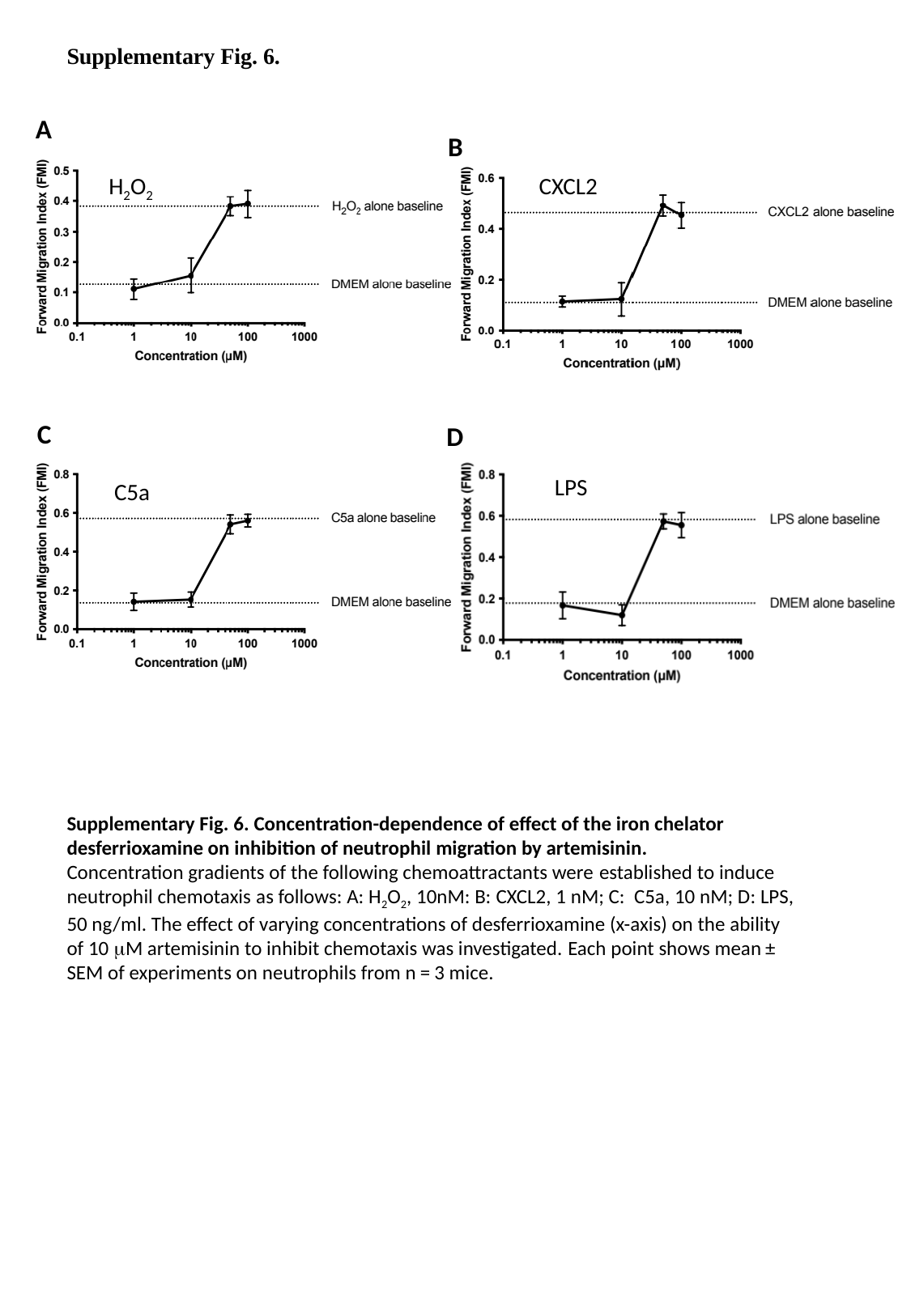

Supplementary Fig. 6.
A
B
CXCL2
H2O2
C
D
LPS
C5a
Supplementary Fig. 6. Concentration-dependence of effect of the iron chelator desferrioxamine on inhibition of neutrophil migration by artemisinin.
Concentration gradients of the following chemoattractants were established to induce neutrophil chemotaxis as follows: A: H2O2, 10nM: B: CXCL2, 1 nM; C: C5a, 10 nM; D: LPS, 50 ng/ml. The effect of varying concentrations of desferrioxamine (x-axis) on the ability of 10 μM artemisinin to inhibit chemotaxis was investigated. Each point shows mean ± SEM of experiments on neutrophils from n = 3 mice.

## Slide 8
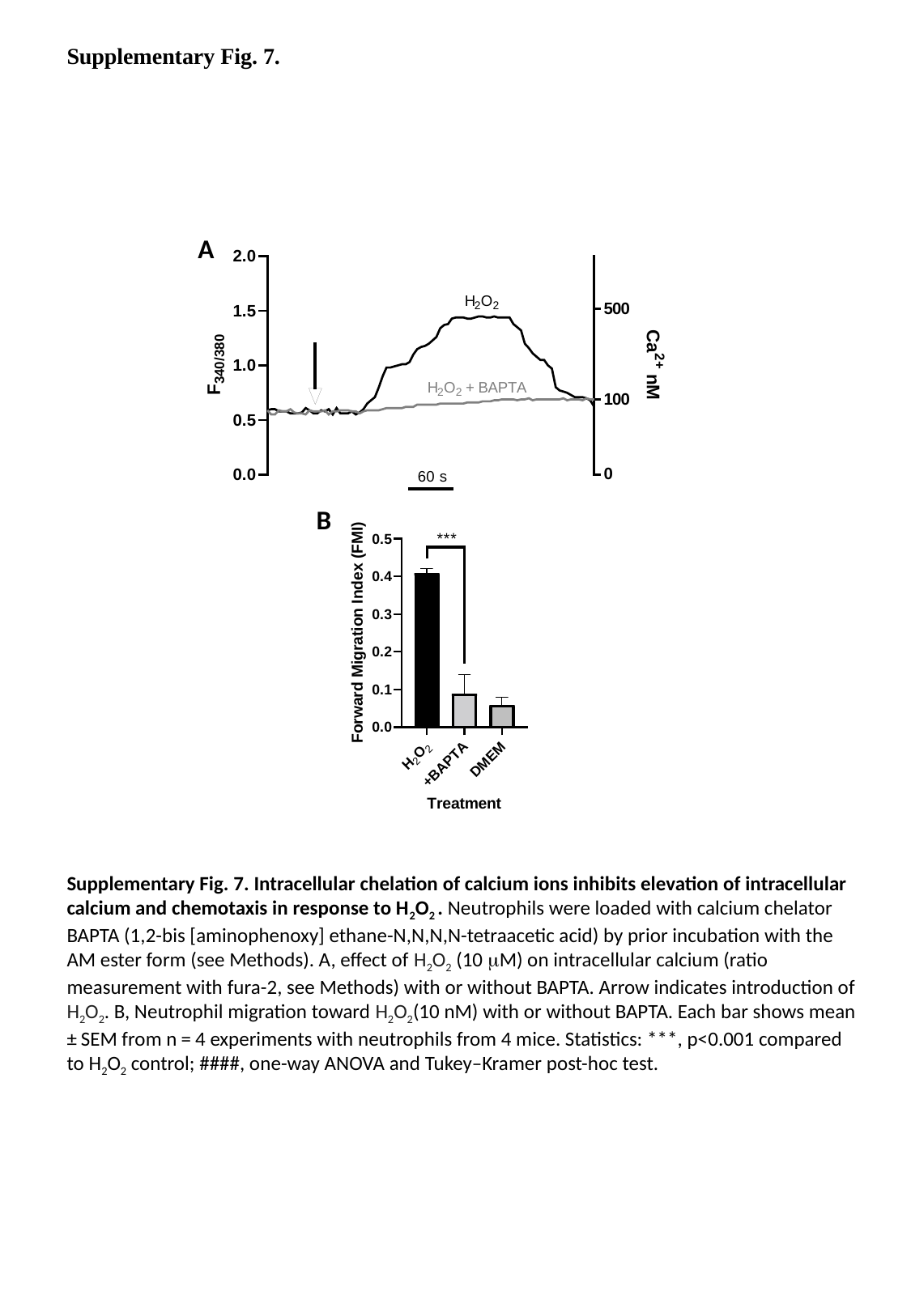

Supplementary Fig. 7.
A
B
Supplementary Fig. 7. Intracellular chelation of calcium ions inhibits elevation of intracellular calcium and chemotaxis in response to H2O2 . Neutrophils were loaded with calcium chelator BAPTA (1,2-bis [aminophenoxy] ethane-N,N,N,N-tetraacetic acid) by prior incubation with the AM ester form (see Methods). A, effect of H2O2 (10 μM) on intracellular calcium (ratio measurement with fura-2, see Methods) with or without BAPTA. Arrow indicates introduction of H2O2. B, Neutrophil migration toward H2O2(10 nM) with or without BAPTA. Each bar shows mean ± SEM from n = 4 experiments with neutrophils from 4 mice. Statistics: ***, p<0.001 compared to H2O2 control; ####, one-way ANOVA and Tukey–Kramer post-hoc test.

## Slide 9
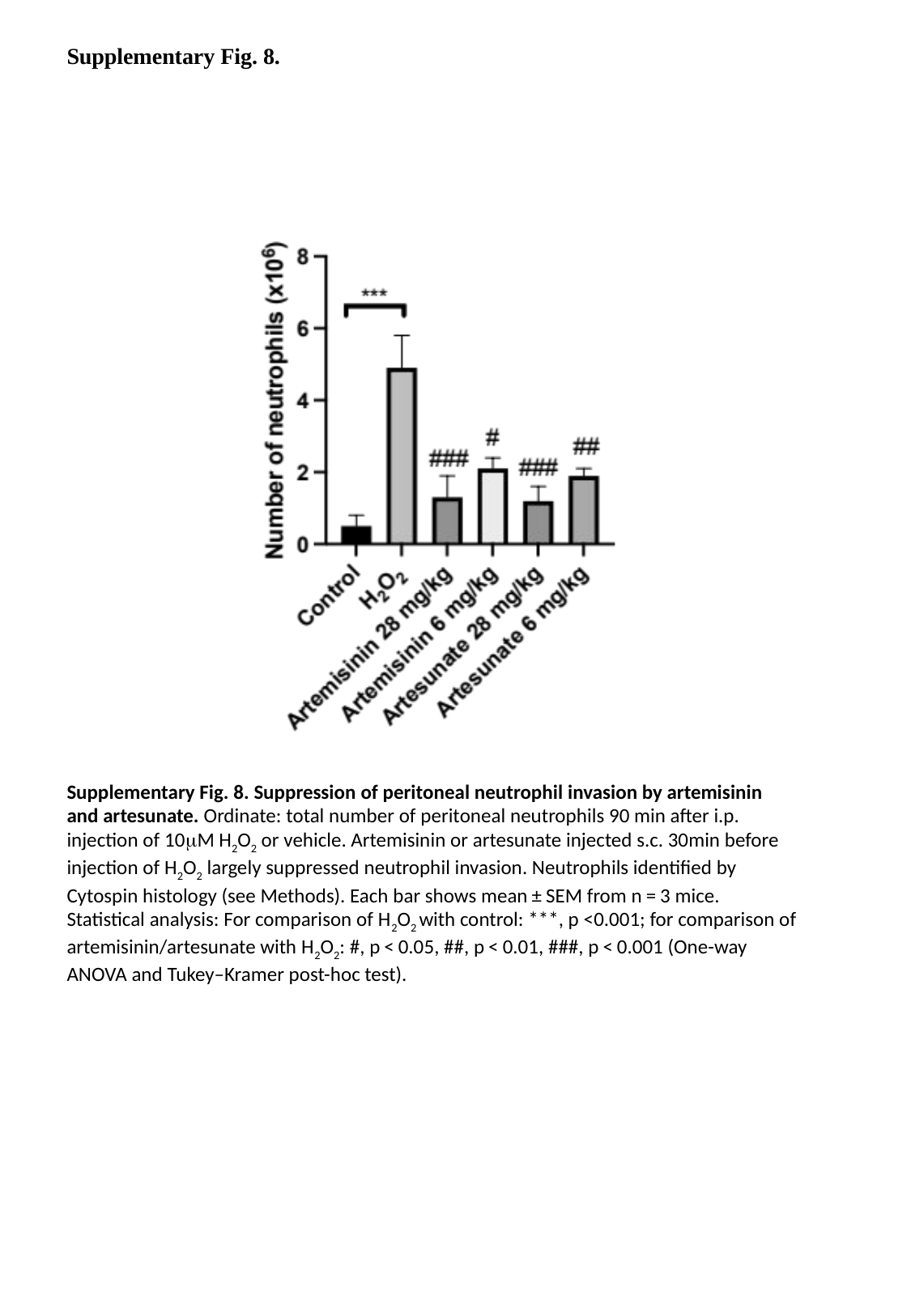

Supplementary Fig. 8.
Supplementary Fig. 8. Suppression of peritoneal neutrophil invasion by artemisinin and artesunate. Ordinate: total number of peritoneal neutrophils 90 min after i.p. injection of 10μM H2O2 or vehicle. Artemisinin or artesunate injected s.c. 30min before injection of H2O2 largely suppressed neutrophil invasion. Neutrophils identified by Cytospin histology (see Methods). Each bar shows mean ± SEM from n = 3 mice. Statistical analysis: For comparison of H2O2 with control: ***, p <0.001; for comparison of artemisinin/artesunate with H2O2: #, p < 0.05, ##, p < 0.01, ###, p < 0.001 (One-way ANOVA and Tukey–Kramer post-hoc test).

## Slide 10
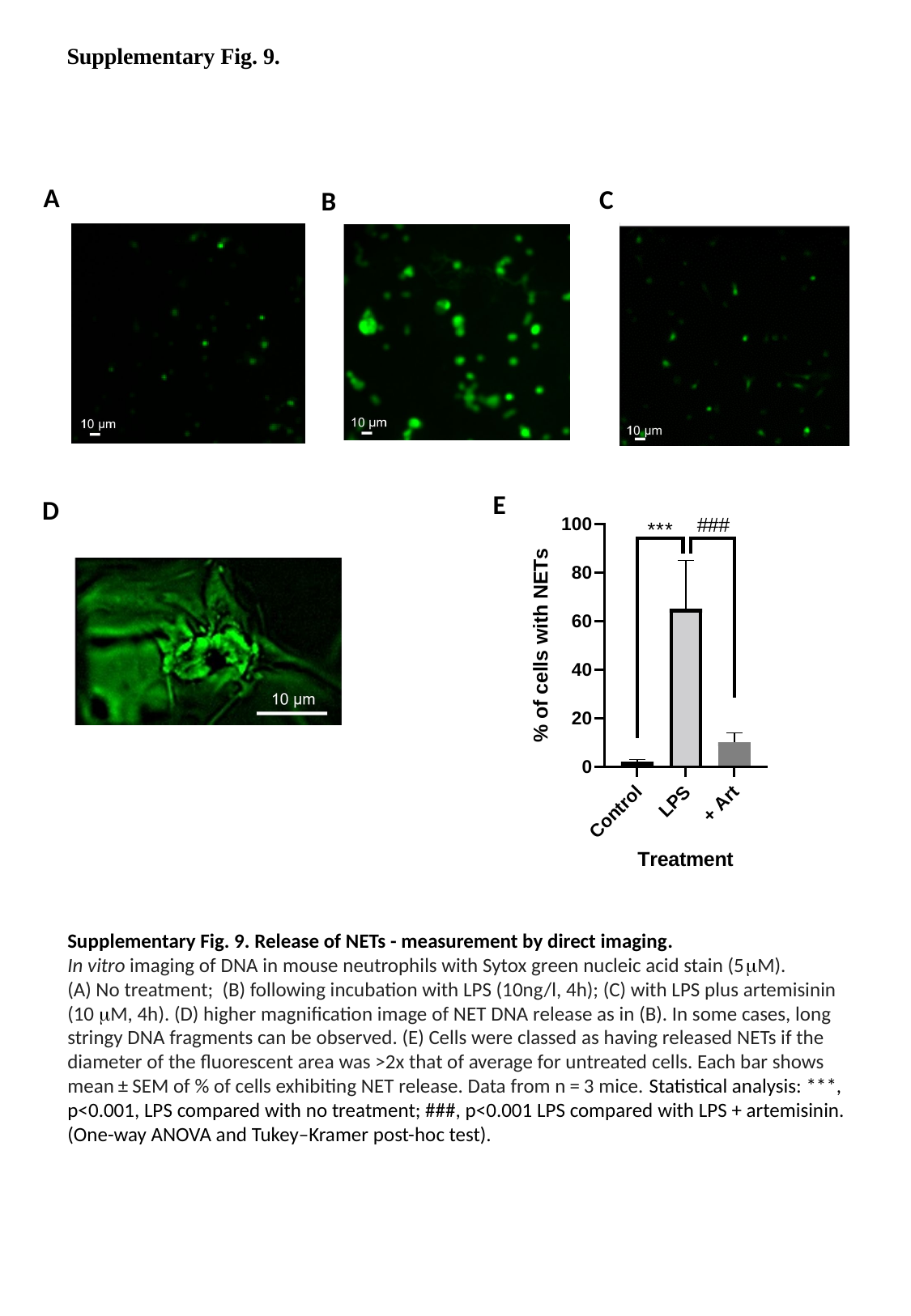

Supplementary Fig. 9.
A
C
B
E
D
Supplementary Fig. 9. Release of NETs - measurement by direct imaging.
In vitro imaging of DNA in mouse neutrophils with Sytox green nucleic acid stain (5μM).
(A) No treatment; (B) following incubation with LPS (10ng/l, 4h); (C) with LPS plus artemisinin (10 μM, 4h). (D) higher magnification image of NET DNA release as in (B). In some cases, long stringy DNA fragments can be observed. (E) Cells were classed as having released NETs if the diameter of the fluorescent area was >2x that of average for untreated cells. Each bar shows mean ± SEM of % of cells exhibiting NET release. Data from n = 3 mice. Statistical analysis: ***, p<0.001, LPS compared with no treatment; ###, p<0.001 LPS compared with LPS + artemisinin. (One-way ANOVA and Tukey–Kramer post-hoc test).

## Slide 11
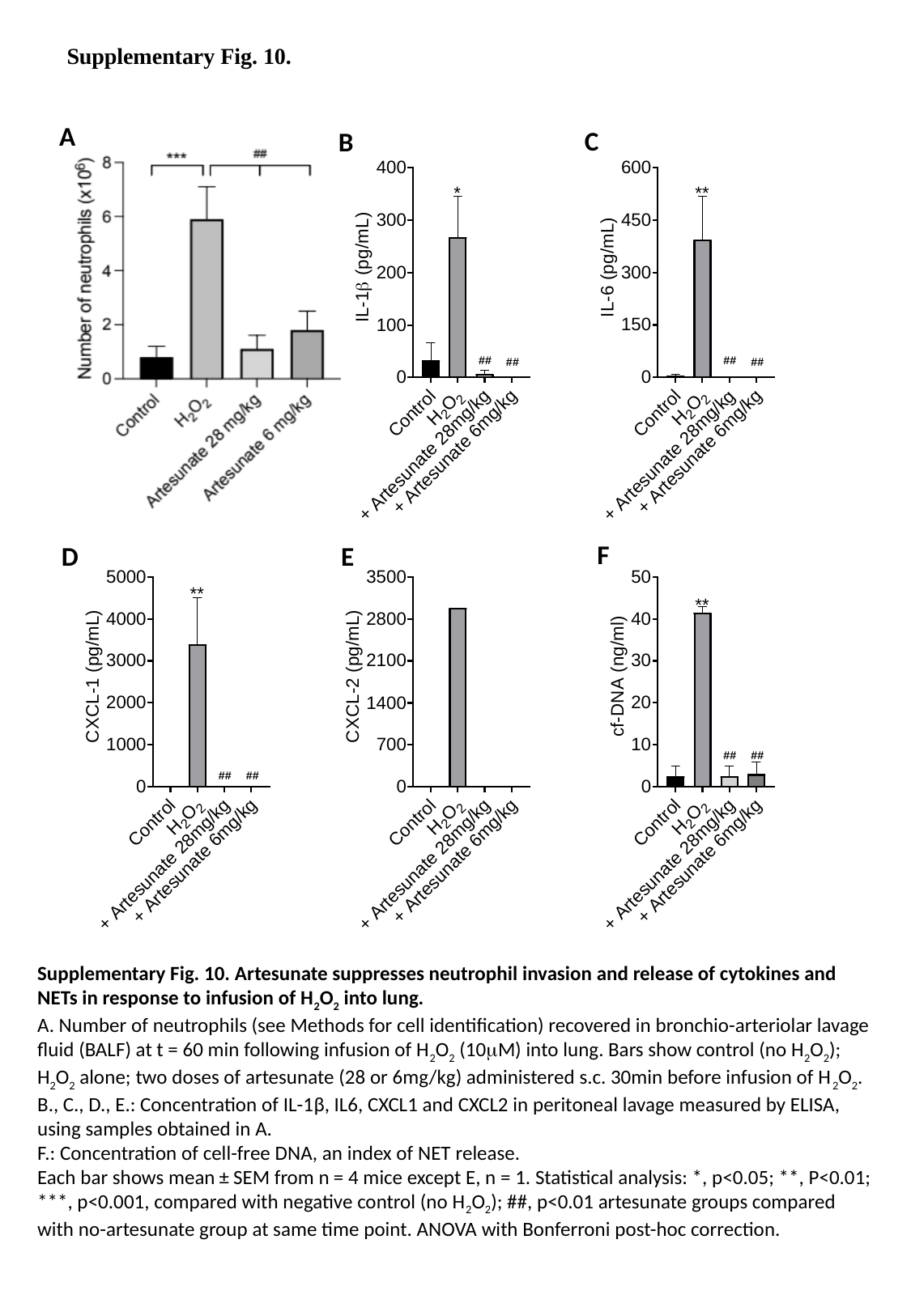

Supplementary Fig. 10.
A
C
B
F
D
E
Supplementary Fig. 10. Artesunate suppresses neutrophil invasion and release of cytokines and NETs in response to infusion of H2O2 into lung.
A. Number of neutrophils (see Methods for cell identification) recovered in bronchio-arteriolar lavage fluid (BALF) at t = 60 min following infusion of H2O2 (10μM) into lung. Bars show control (no H2O2); H2O2 alone; two doses of artesunate (28 or 6mg/kg) administered s.c. 30min before infusion of H2O2.
B., C., D., E.: Concentration of IL-1β, IL6, CXCL1 and CXCL2 in peritoneal lavage measured by ELISA, using samples obtained in A.
F.: Concentration of cell-free DNA, an index of NET release.
Each bar shows mean ± SEM from n = 4 mice except E, n = 1. Statistical analysis: *, p<0.05; **, P<0.01; ***, p<0.001, compared with negative control (no H2O2); ##, p<0.01 artesunate groups compared with no-artesunate group at same time point. ANOVA with Bonferroni post-hoc correction.

## Slide 12
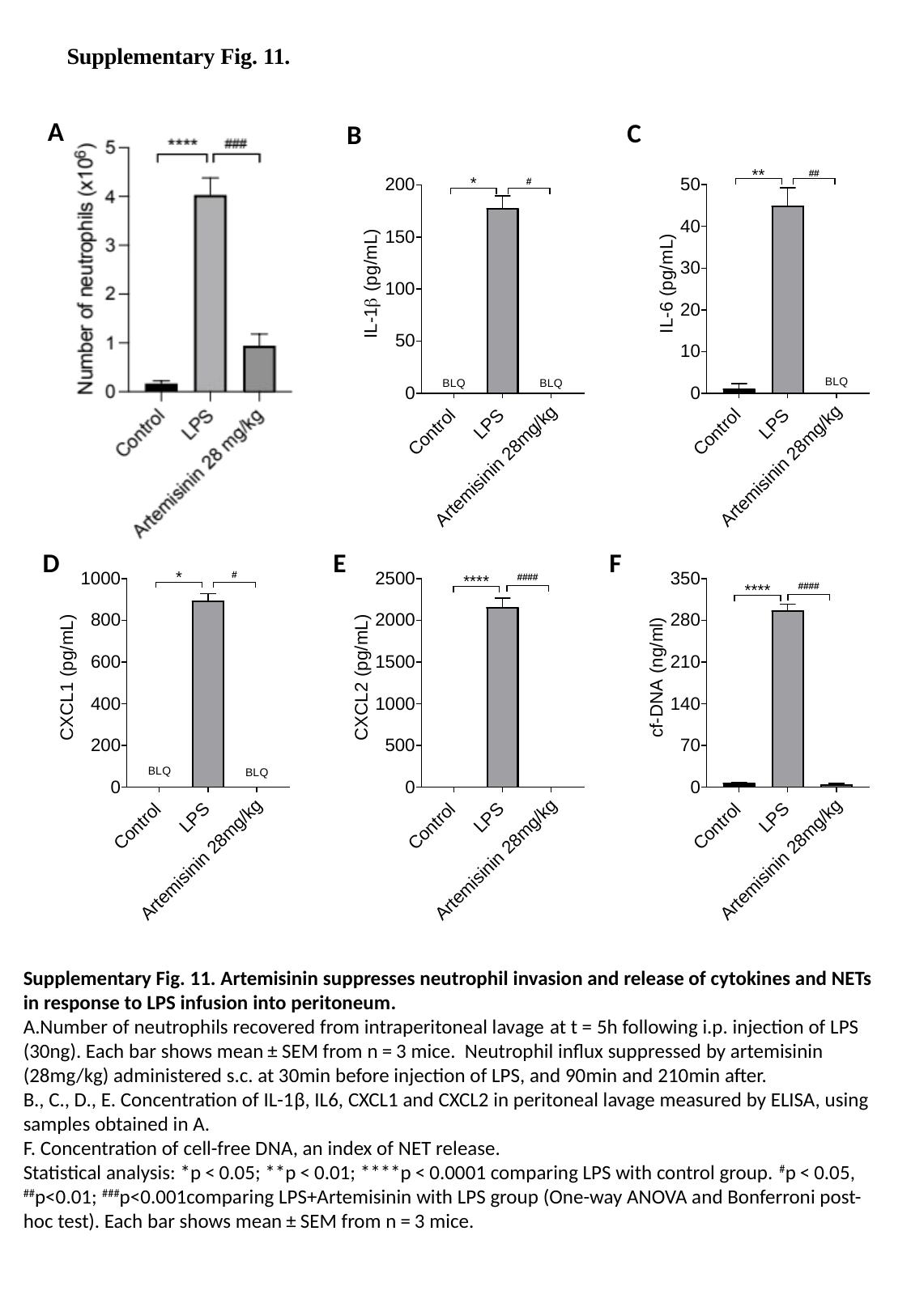

Supplementary Fig. 11.
A
C
B
D
E
F
Supplementary Fig. 11. Artemisinin suppresses neutrophil invasion and release of cytokines and NETs in response to LPS infusion into peritoneum.
Number of neutrophils recovered from intraperitoneal lavage at t = 5h following i.p. injection of LPS (30ng). Each bar shows mean ± SEM from n = 3 mice. Neutrophil influx suppressed by artemisinin (28mg/kg) administered s.c. at 30min before injection of LPS, and 90min and 210min after.
B., C., D., E. Concentration of IL-1β, IL6, CXCL1 and CXCL2 in peritoneal lavage measured by ELISA, using samples obtained in A.
F. Concentration of cell-free DNA, an index of NET release.
Statistical analysis: *p < 0.05; **p < 0.01; ****p < 0.0001 comparing LPS with control group. #p < 0.05, ##p<0.01; ###p<0.001comparing LPS+Artemisinin with LPS group (One-way ANOVA and Bonferroni post-hoc test). Each bar shows mean ± SEM from n = 3 mice.

## Slide 13
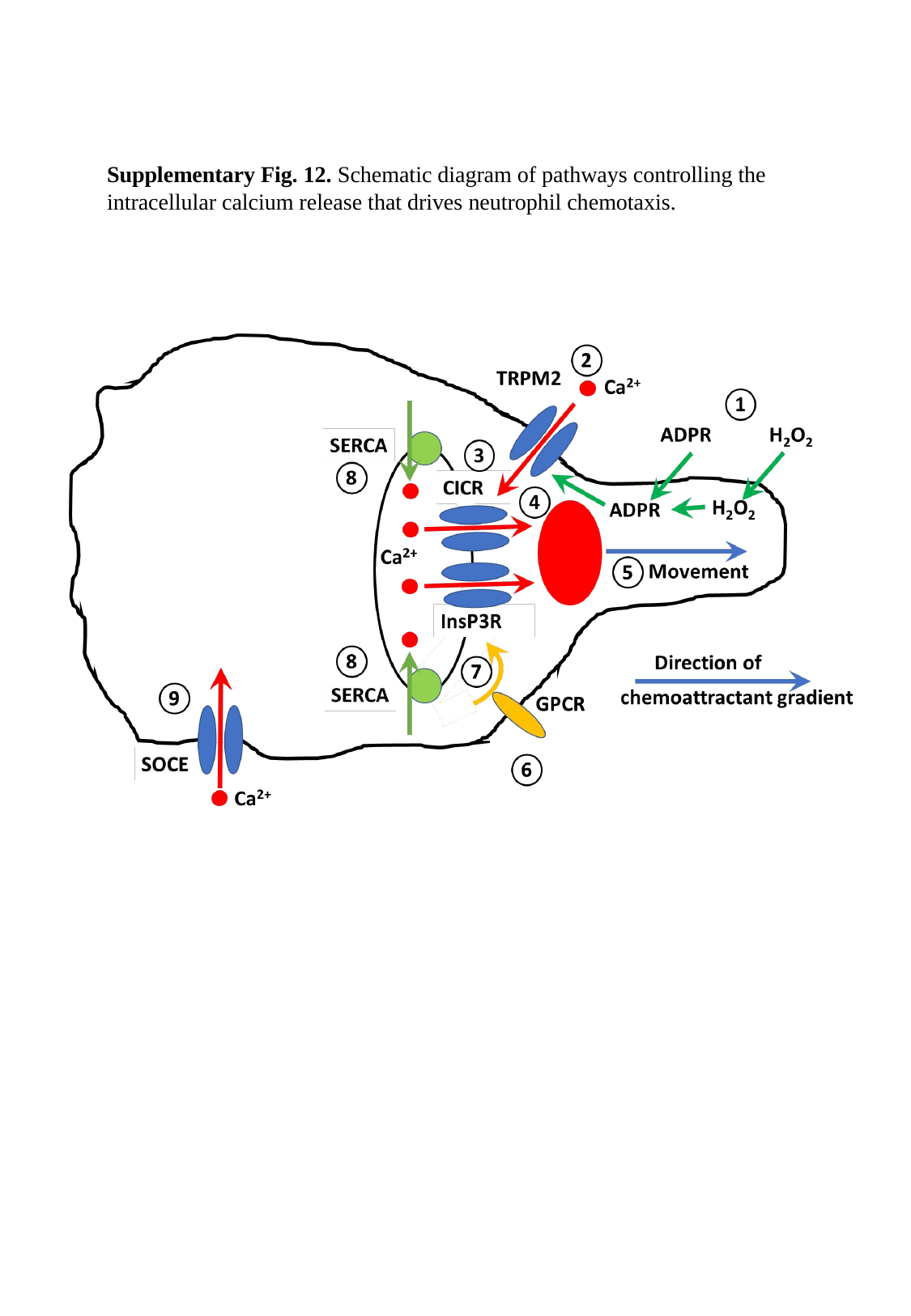

Supplementary Fig. 12. Schematic diagram of pathways controlling the intracellular calcium release that drives neutrophil chemotaxis.

## Slide 14
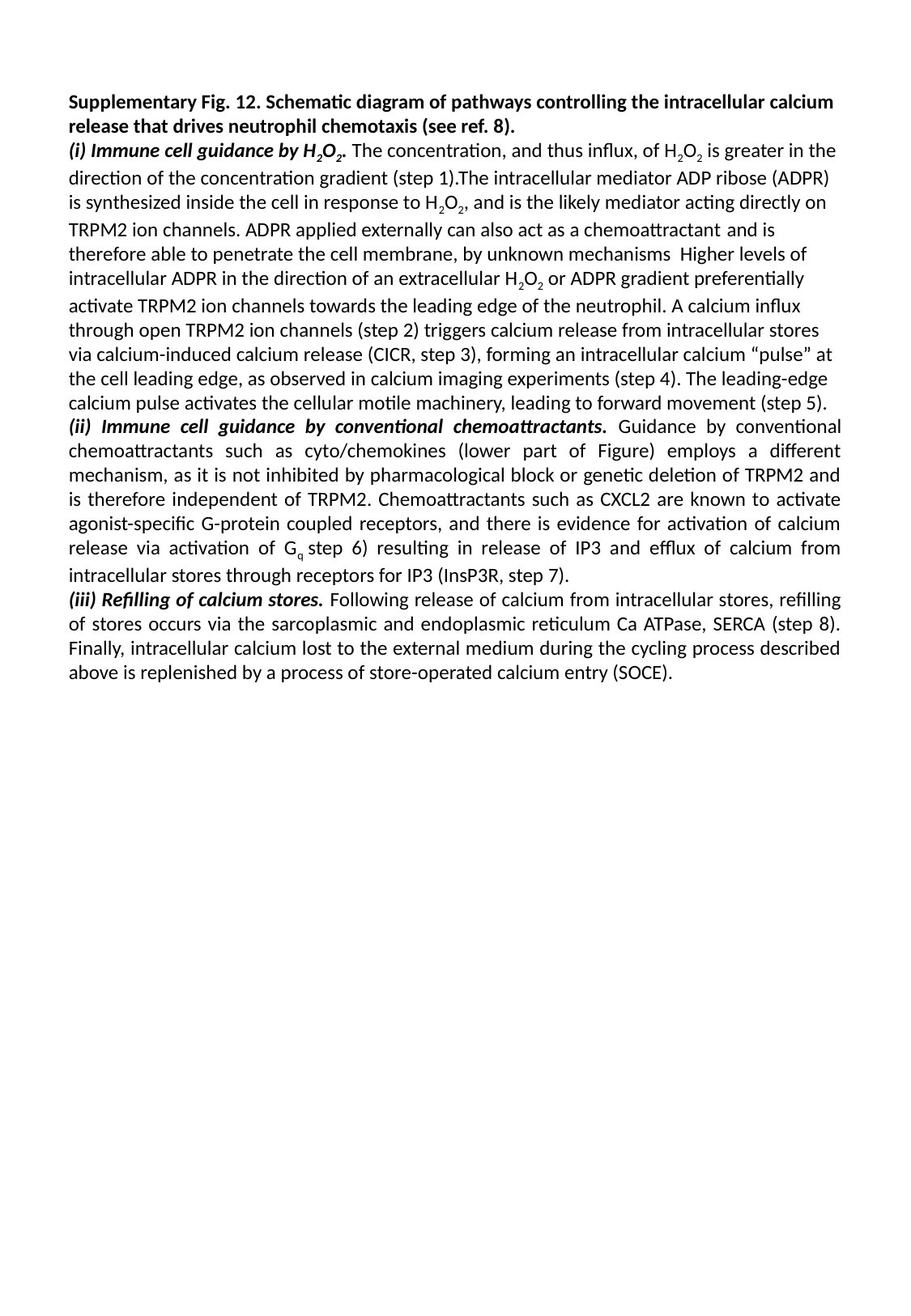

Supplementary Fig. 12. Schematic diagram of pathways controlling the intracellular calcium release that drives neutrophil chemotaxis (see ref. 8).
(i) Immune cell guidance by H2O2. The concentration, and thus influx, of H2O2 is greater in the direction of the concentration gradient (step 1).The intracellular mediator ADP ribose (ADPR) is synthesized inside the cell in response to H2O2, and is the likely mediator acting directly on TRPM2 ion channels. ADPR applied externally can also act as a chemoattractant and is therefore able to penetrate the cell membrane, by unknown mechanisms Higher levels of intracellular ADPR in the direction of an extracellular H2O2 or ADPR gradient preferentially activate TRPM2 ion channels towards the leading edge of the neutrophil. A calcium influx through open TRPM2 ion channels (step 2) triggers calcium release from intracellular stores via calcium-induced calcium release (CICR, step 3), forming an intracellular calcium “pulse” at the cell leading edge, as observed in calcium imaging experiments (step 4). The leading-edge calcium pulse activates the cellular motile machinery, leading to forward movement (step 5).
(ii) Immune cell guidance by conventional chemoattractants. Guidance by conventional chemoattractants such as cyto/chemokines (lower part of Figure) employs a different mechanism, as it is not inhibited by pharmacological block or genetic deletion of TRPM2 and is therefore independent of TRPM2. Chemoattractants such as CXCL2 are known to activate agonist-specific G-protein coupled receptors, and there is evidence for activation of calcium release via activation of Gq step 6) resulting in release of IP3 and efflux of calcium from intracellular stores through receptors for IP3 (InsP3R, step 7).
(iii) Refilling of calcium stores. Following release of calcium from intracellular stores, refilling of stores occurs via the sarcoplasmic and endoplasmic reticulum Ca ATPase, SERCA (step 8). Finally, intracellular calcium lost to the external medium during the cycling process described above is replenished by a process of store-operated calcium entry (SOCE).
