# Supplementary material for: Artemisinin inhibits neutrophil and macrophage chemotaxis, cytokine production and NET release
Source: Sci Rep. 2022 Jun 30;12:11078. doi: 10.1038/s41598-022-15214-6 (PMC9245885; doi:10.1038/s41598-022-15214-6)
Supplement: Supplementary file 2 — Supplementary Information 2. [file 41598_2022_15214_MOESM2_ESM.pptx]

## Slide 1
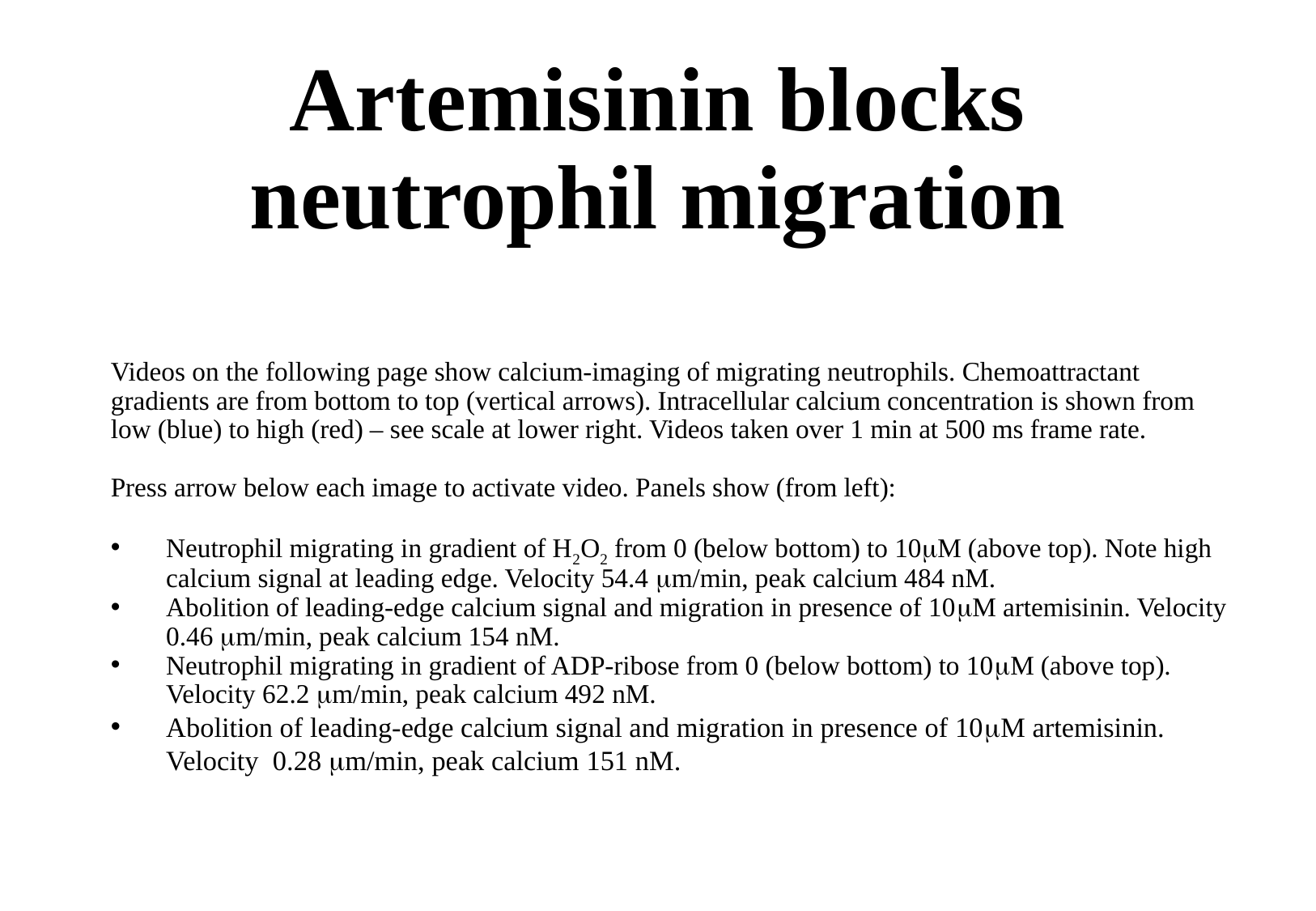

# Artemisinin blocks neutrophil migration
Videos on the following page show calcium-imaging of migrating neutrophils. Chemoattractant gradients are from bottom to top (vertical arrows). Intracellular calcium concentration is shown from low (blue) to high (red) – see scale at lower right. Videos taken over 1 min at 500 ms frame rate.
Press arrow below each image to activate video. Panels show (from left):
Neutrophil migrating in gradient of H2O2 from 0 (below bottom) to 10μM (above top). Note high calcium signal at leading edge. Velocity 54.4 μm/min, peak calcium 484 nM.
Abolition of leading-edge calcium signal and migration in presence of 10μM artemisinin. Velocity 0.46 μm/min, peak calcium 154 nM.
Neutrophil migrating in gradient of ADP-ribose from 0 (below bottom) to 10μM (above top). Velocity 62.2 μm/min, peak calcium 492 nM.
Abolition of leading-edge calcium signal and migration in presence of 10μM artemisinin. Velocity 0.28 μm/min, peak calcium 151 nM.

## Slide 2
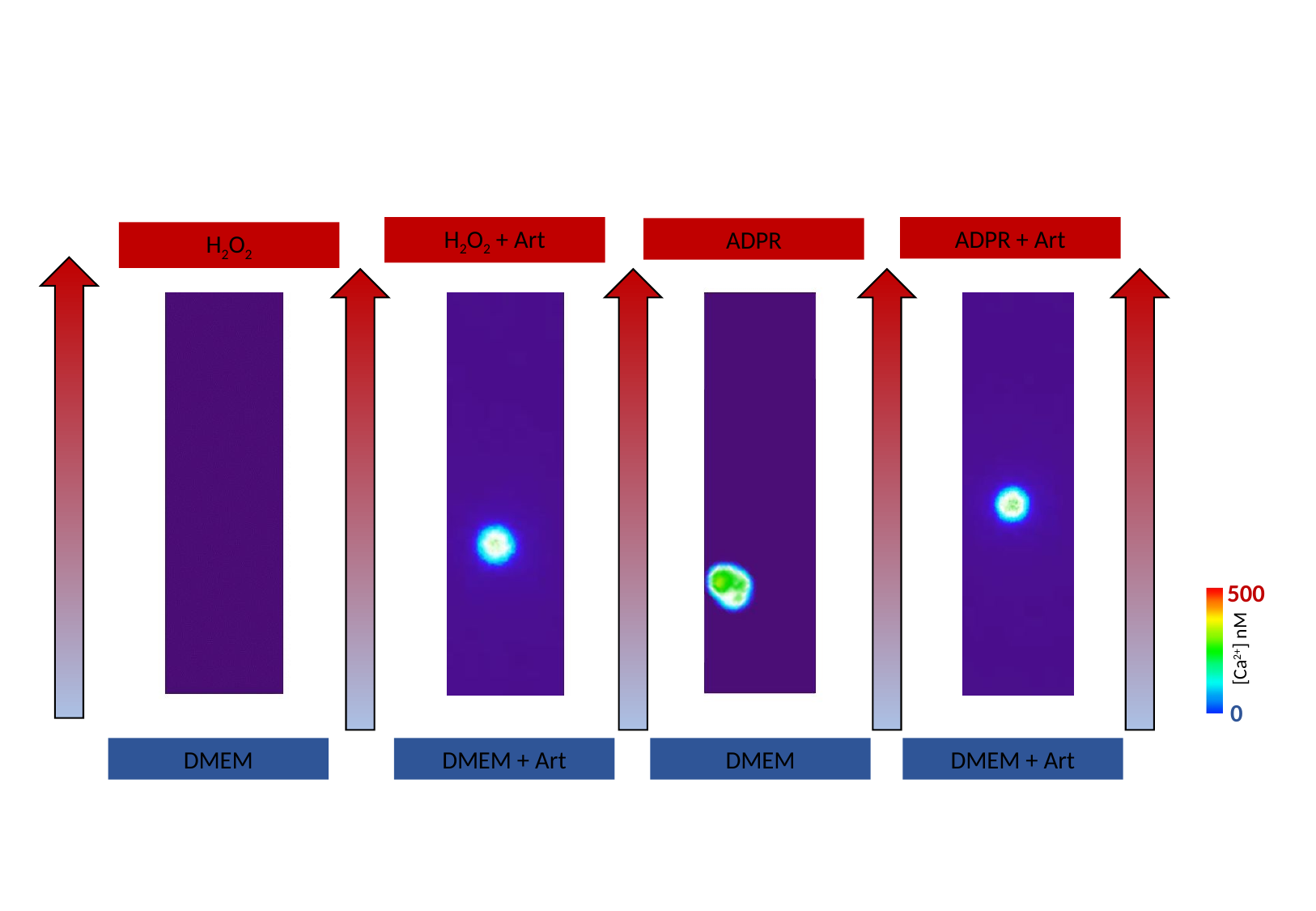

H2O2 + Art
ADPR + Art
ADPR
H2O2
500
[Ca2+] nM
0
DMEM
DMEM + Art
DMEM
DMEM + Art
